# Honokiol Prevents Non-Alcoholic Steatohepatitis-Induced Liver Cancer via EGFR Degradation through the Glucocorticoid Receptor—MIG6 Axis

**DOI:** 10.3390/cancers13071515

**Published:** 2021-03-25

**Authors:** Keiichiro Okuda, Atsushi Umemura, Shiori Umemura, Seita Kataoka, Hiroyoshi Taketani, Yuya Seko, Taichiro Nishikawa, Kanji Yamaguchi, Michihisa Moriguchi, Yoshihiro Kanbara, Jack L. Arbiser, Toshihide Shima, Takeshi Okanoue, Michael Karin, Yoshito Itoh

**Affiliations:** 1 Department of Gastroenterology and Hepatology, Kyoto Prefectural University of Medicine, 465 Kajii-cho, Kamigyo-ku, Kyoto 602-8566, Japan; k-okuda@koto.kpu-m.ac.jp (K.O.); s1120@koto.kpu-m.ac.jp (S.K.); take1012@koto.kpu-m.ac.jp (H.T.); yuyaseko@koto.kpu-m.ac.jp (Y.S.); taichi@koto.kpu-m.ac.jp (T.N.); ykanji@koto.kpu-m.ac.jp (K.Y.); mmori@koto.kpu-m.ac.jp (M.M.); yitoh@koto.kpu-m.ac.jp (Y.I.); 2 Department of Obstetrics and Gynecology, Graduate School of Medical Science, Kyoto Prefectural University of Medicine, 465 Kajii-cho, Kamigyo-ku, Kyoto 602-8566, Japan; sumemura@koto.kpu-m.ac.jp; 3 Department of Gastroenterology and Hepatology, Saiseikai Suita Hospital, Suita 564-0013, Japan; kanbara1949d@suita.saiseikai.or.jp (Y.K.); shima0301d@suita.saiseikai.or.jp (T.S.); okanoue@suita.saiseikai.or.jp (T.O.); 4 Department of Dermatology, Emory University School of Medicine, Atlanta, GA 30322, USA; jarbise@emory.edu; 5 Veterans Affairs Medical Center, Decatur, GA 30322, USA; 6 Laboratory of Gene Regulation and Signal Transduction, Departments of Pharmacology, School of Medicine, University of California San Diego, 9500 Gilman Drive, La Jolla, San Diego, CA 92093, USA; mkarin@health.ucsd.edu; 7 Departments of Pathology, School of Medicine, University of California San Diego, 9500 Gilman Drive, La Jolla, San Diego, CA 92093, USA

**Keywords:** liver cancer, glucocorticoid receptor, MIG-6/ERRFI1, epidermal growth factor receptor (EGFR), honokiol

## Abstract

**Simple Summary:**

Non-alcoholic fatty liver disease (NAFLD) is a major health problem globally linked with the growing prevalence of metabolic syndrome. A subset of patients with NAFLD progress to non-alcoholic steatohepatitis (NASH), which increases the risk of hepatocellular carcinoma (HCC). However, the mechanisms responsible for the progression to HCC are unclear, and no preventative modalities have been developed. To address this issue, the present study used the natural compound honokiol to clarify the mechanism of this process. The results illustrated that epidermal growth factor receptor (EGFR) was upregulated in mice with NASH, and treatment with honokiol inhibited EGFR and the progression to HCC. Further analysis illustrated that honokiol increased glucocorticoid receptor (GR) nuclear translocation and mitogen-inducible gene 6 (MIG6)/ERBB receptor feedback inhibitor 1 (ERRFI1) expression, thereby promoting EGFR degradation. These findings were confirmed in tissues from patients with NASH and HCC.

**Abstract:**

Non-alcoholic steatohepatitis (NASH) has become a serious public health problem associated with metabolic syndrome. The mechanisms by which NASH induces hepatocellular carcinoma (HCC) remain unknown. There are no approved drugs for treating NASH or preventing NASH-induced HCC. We used a genetic mouse model in which HCC was induced via high-fat diet feeding. This mouse model strongly resembles human NASH-induced HCC. The natural product honokiol (HNK) was tested for its preventative effects against NASH progression to HCC. Then, to clarify the mechanisms underlying HCC development, human HCC cells were treated with HNK. Human clinical specimens were also analyzed to explore this study’s clinical relevance. We found that epidermal growth factor receptor (EGFR) signaling was hyperactivated in the livers of mice with NASH and human HCC specimens. Inhibition of EGFR signaling by HNK drastically attenuated HCC development in the mouse model. Mechanistically, HNK accelerated the nuclear translocation of glucocorticoid receptor (GR) and promoted mitogen-inducible gene 6 (MIG6)/ERBB receptor feedback inhibitor 1 (ERRFI1) expression, leading to EGFR degradation and thereby resulting in robust tumor suppression. In human samples, EGFR-positive HCC tissues and their corresponding non-tumor tissues exhibited decreased *ERRFI1* mRNA expression. Additionally, GR-positive non-tumor liver tissues displayed lower EGFR expression. Livers from patients with advanced NASH exhibited decreased *ERRFI1* expression. EGFR degradation or inactivation represents a novel approach for NASH–HCC treatment and prevention, and the GR–MIG6 axis is a newly defined target that can be activated by HNK and related compounds.

## 1. Introduction

Non-alcoholic fatty liver disease (NAFLD) is a serious public health problem associated with the global increase in the incidence of metabolic syndrome, which comprises type 2 diabetes, dyslipidemia, and hypertension. NAFLD is extremely common with a global prevalence of 25.2% [1]. A subgroup of patients with NAFLD progress to non-alcoholic steatohepatitis (NASH), which is a more severe disease associated with liver damage, inflammation, and fibrosis [1]. In addition, NASH greatly increases the risk of HCC. It is reported that the annual incidence rate for HCC in patients with cirrhotic NASH was 2.6% in the United States [2], and the 5-year HCC rate in such patients was 11.3% in Japan [3].

Despite being an important cause of liver cancer, the precise mechanisms by which NASH progresses to HCC remain unknown. Because the pathophysiology of NASH is heterogeneous and complex, it has been difficult to develop widely applicable NASH–HCC drugs, especially for disease prevention. Correspondingly, the only strategy for preventing the progression of early NAFLD to NASH is lifestyle modification, including dieting and exercise. Because almost all HCCs arise from chronic and common liver diseases, including NASH, the effective prevention of this common cancer should rely on the application of safe and low-cost drugs, including naturally occurring compounds.

Epidermal growth factor receptor (EGFR) is a member of the ErbB subfamily of receptor type tyrosine kinases. In response to binding EGF and other ligands, EGFR stimulates cell proliferation via the RAS/RAF/ERK module, mechanistic (or mammalian) target of rapamycin (mTOR) (predominantly mTOR complex 1 (mTORC1)), and other effectors [4]. EGFR gene-activating mutations have been detected in a variety of cancers, including lung, colorectal, head and neck, and pancreatic cancers [4]. However, mutational activation of EGFR signaling is relatively uncommon in HCC [5,6], and correspondingly, EGFR inhibitors has not been effective against HCC [7].

EGFR, together with MET, is a major regulator of liver regeneration and hepatocyte proliferation [8,9]. It was recently reported that pharmacologic inhibition of EGFR suppresses NAFLD [10,11], and MET is not involved in NAFLD development. EGFR is a uniquely important tyrosine kinase that could be a strong potential target for NASH prevention.

Nonetheless, the mechanism by which EGFR influences NAFLD/NASH-induced HCC progression remains unclear. Because liver cancer arises from chronic liver damage, EGFR signaling may be needed for the maintenance of liver function. Therefore, strong and direct EGFR inhibition may exacerbate liver damage, and this approach should be attempted cautiously.

In the present study, we searched for novel modalities to prevent NAFLD/NASH-driven HCC via EGFR suppression using naturally occurring compounds. We found that honokiol (HNK) can prevent HCC development in a NAFLD mouse model induced by diethylnitrosamine (DEN) treatment and high-fat diet (HFD) feeding and in a major urinary protein (MUP)-urokinase type plasminogen activator (uPA) transgenic NASH mouse model [12,13].

HNK has been reported to have anti-tumor properties, but its effects on NAFLD/NASH-driven HCC have not been thoroughly examined. We demonstrated that HNK exerts preventative effects on HCC development in NAFLD/NASH mouse models by suppressing EGFR through a degradation mechanism.

## 2. Results

### 2.1. HNK Treatment Attenuates HCC Development in MUP-uPA Mice

To prevent hepatocarcinogenesis in a chronically injured liver, non-toxic naturally occurring compounds or herbal drugs are likely to be more suitable than cytotoxic anticancer drugs that further reduce liver function. To this end, we focused on HNK a bioactive compound extracted from the tree *Magnolia grandiflora* that has been widely used as a constituent of herbal drugs with anti-tumor properties [14]. It was also reported that HNK exhibited suppressive effects in an HCC metastasis model and in a xenograft model using HepG2 cells [15].

**Figure 1:**
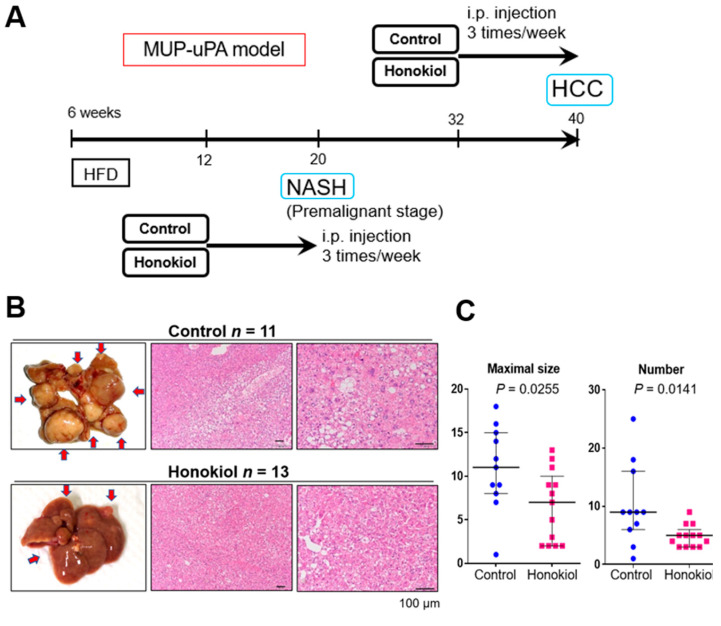

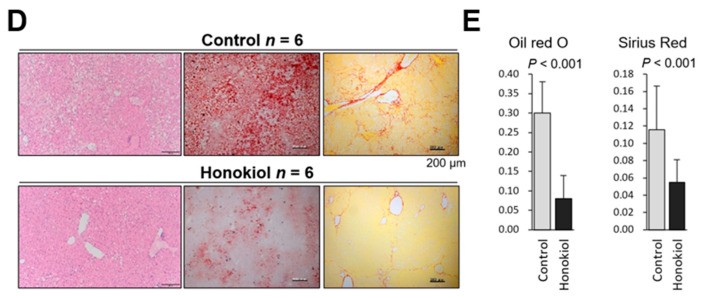
Honokiol (HNK) treatment attenuates hepatocellular carcinoma (HCC) development in major urinary protein (MUP)-urokinase type plasminogen activator (uPA) transgenic mice. (**A**) Protocol for honokiol treatment in MUP-uPA transgenic mice fed a high-fat diet (HFD). Then, six week-old male mice fed an HFD for 26 weeks were treated with vehicle control or HNK (10 mg/kg, intraperitoneally (three times/week) for another 8 weeks (32–40 weeks of age). Tumor development was analyzed at 40 weeks. For the non-alcoholic steatohepatitis (NASH) analysis, 6-week-old male mice fed an HFD for 6 weeks were treated with vehicle or HNK for 8 weeks (12–20 weeks of age). During honokiol treatment, the HFD feeding regimen was continued. (**B**) Gross morphology of livers with HCC and typical HCC histology in mice of the MUP-HFD model. (**C**) Maximal tumor size and tumor numbers in MUP-HFD mice treated with vehicle or honokiol. Tumor development was analyzed 2–4 days after the final honokiol injection. Results are presented as the median with interquartile ranges. (**D**) Liver sections of MUP-HFD mice were analyzed at 20 weeks of age after treatment with vehicle or honokiol for the last 8 weeks of NASH development. Liver histology, lipid accumulation, and fibrosis were analyzed by staining liver sections with hematoxylin and eosin, Oil red O, and Sirius Red, respectively. (**E**) The positive areas were quantified using ImageJ software and presented as bar graphs. Results are presented as the mean ± SD.

We examined the ability of HNK to inhibit the progression from NASH-to-HCC in the MUP-uPA model. In response to HFD feeding from 6 to 40 weeks of age, MUP-uPA mice first develop NASH and then robustly (85–90% penetrance) progress to HCC. In the present study, we intraperitoneally injected HNK or vehicle into MUP-uPA mice fed an HFD three times per week from 32 to 40 weeks of age, the last 2 months of HCC development (Figure 1A). As expected, mice in both groups developed typical HCCs, including the steatohepatitic type at 40 weeks (Figure 1B). Notably, HNK treatment significantly reduced both the tumor maximal size and multiplicity (Figure 1C). HNK injection into the mice from 32 to 40 weeks of age did not cause body weight reduction or liver weight loss (Figure S1A). Notably, HNK attenuated liver injury, suggesting that the treatment could suppress NASH development (Figure S1A).

To explore whether HNK inhibits NASH development in MUP-uPA mice, we injected HNK or vehicle into HFD-fed MUP-uPA mice three times per week from 12 to 20 weeks of age, the last 2 months of NASH development (Figure 1A). As expected, HCC did not develop in the MUP-uPA mice at 20 weeks of age regardless of the treatment. HNK treatment significantly attenuated steatosis and fibrosis of the liver (Figure 1D). HNK treatment decreased body weight gain and attenuated liver injury (Figure S1B).

These results suggest that HNK exerts anti-HCC effects in the NASH mouse model and attenuates NASH development [12].

### 2.2. EGFR Signaling Is Upregulated in the HFD-fed MUP-uPA Mouse Liver

The mechanism by which HNK attenuates hepatocarcinogenesis in MUP-uPA mice was investigated. Although HNK treatment at the early stage suppressed the development of NASH in MUP-uPA mice, HNK treatment for the last 8 weeks of HCC development clarified its anti-tumor effects independently, at least in part of NASH improvement.

According to previous findings, the MUP-uPA mice model is based on the spontaneous development of tumors after long-lasting liver injuries [13,16]. This observation indicates that long-term chronic liver damage in this mouse model activates mechanisms of mutagenesis similar to those observed in patients with chronic liver disease. HCC in HFD-fed MUP-uPA mice carries numerous non-recurrent mutations, suggesting that pre-cancerous hepatocytes may acquire oncogenic properties before HCC arises.

**Figure 2:**
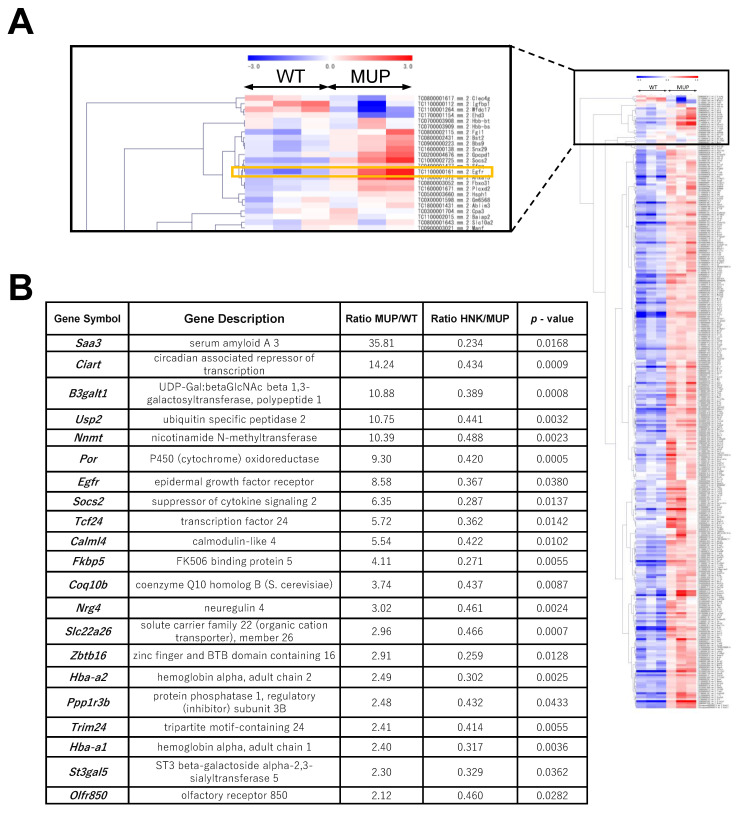
Epidermal growth factor receptor (EGFR) signaling is upregulated in HFD-fed MUP-uPA mouse liver. (**A**) Hierarchical clustering results of differentially expressed genes between HFD-fed MUP-uPA (MUP) and HFD-fed wild-type B6 (WT) mouse livers (*n* = 3 per group). The list of genes analyzed here is shown in Table S1. (**B**) A total of 21 genes, including *Egfr* and *Fkbp5*, were upregulated in MUP-uPA mice, and suppressed by HNK-treatment.

To examine the effects of HNK on oncogenic signaling in the background liver, we conducted RNA array analysis of non-tumor liver tissues extracted from 40-week-old mice harboring HCC. Among >20,000 well-annotated genes, 288 genes were upregulated by ≥2.0 in non-tumor liver tissue from HFD-fed MUP-uPA mice (MUP) compared with the findings in HFD-fed wild-type (WT) mice (Figure 2A and Table S1). In all, 21 genes including *Egfr* (also known as *Erbb*) and FK506-binding protein 5 (*Fkbp5*) were upregulated ≥2.0-fold in the murine NASH liver and were downregulated ≥0.5-fold by HNK treatment (*p*-value < 0.05, Figure 2B).

Notably, FKBP5 is an oncogenic molecular chaperon and oncoprotein that inhibits GR activation [17].

### 2.3. HNK Treatment Suppresses EGFR Signaling in the NASH Liver

**Figure 3:**
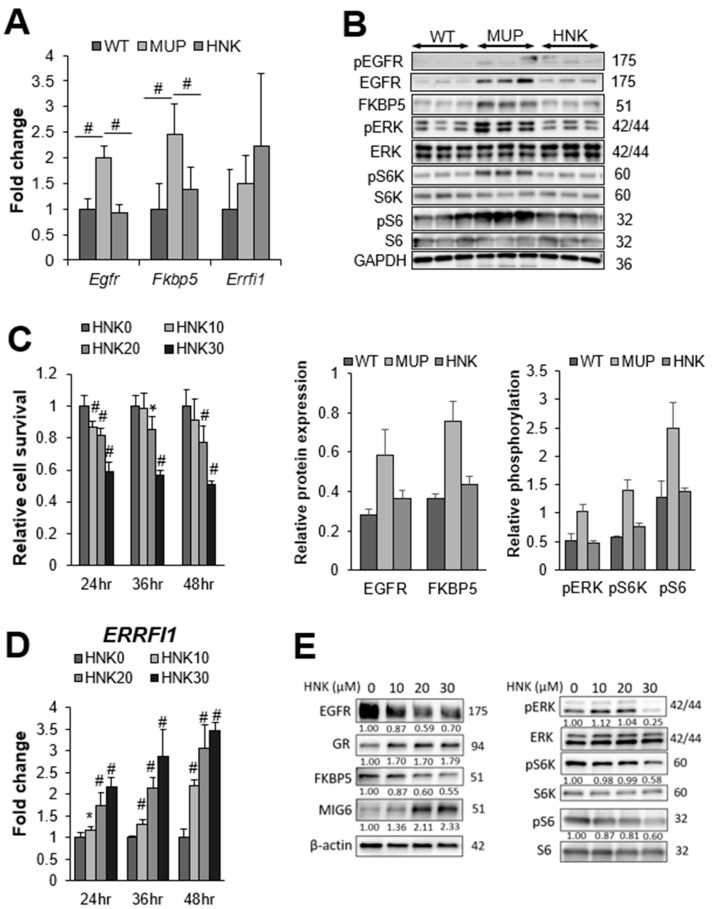
Honokiol (HNK) treatment suppresses epidermal growth factor receptor (EGFR) signaling in the non-alcoholic steatohepatitis (NASH) liver. (**A**) Relative expression of *Egfr*, *FK506 binding protein 5 (Fkbp5),* and *ERBB receptor feedback inhibitor 1 (ERRFI1)* mRNAs in non-tumor livers from major urinary protein (MUP)-urokinase type plasminogen activator (uPA) transgenic mice receiving vehicle control (MUP) or HNK and their wild-type (WT) littermates receiving vehicle control (*n* = 5 per group). All mice were fed a high-fat diet (HFD) from 6 weeks of age until sacrifice at 40 weeks of age. Results are presented as the mean ± SD (# *p* < 0.01). (**B**) Immunoblot analysis of the non-tumor liver extracts from WT, MUP, and HNK mice. Protein expression and phosphorylation of EGFR signaling-related molecules, and FKBP5 are presented (*n* = 3 per group). Glyceraldehyde-3-phosphate dehydrogenase (GAPDH) was used as a loading control. (**C**) Relative cell viability and proliferation, and (**D**) relative *ERRFI1* mRNA expression in Hep3B cells 24, 36, and 48 h after incubation with 0, 10, 20, or 30 μM HNK. All graphs represent the mean ± SD (* *p* < 0.05, # *p* < 0.01 vs. HNK0 condition at the same time course). (**E**) Immunoblot analysis of Hep3B cells 24 h after incubation with the indicated concentrations (μM) of HNK. Protein expression and phosphorylation of EGFR signaling-related molecules are presented. β-actin was used as a loading control.

Next, we confirmed the mRNA expression of *Egfr* and *Fkbp5* via real-time qPCR. Consistent with the RNA array data, both *Egfr* and *Fkbp5* were significantly upregulated in HFD-fed MUP-uPA livers compared with their expression in WT livers. Both *Egfr* and *Fkbp5* mRNA expressions were suppressed by HNK treatment (Figure 3A). EGFR and FKBP5 protein expressions were also upregulated in MUP-uPA livers but not in WT background livers (Figure 3B). Although the increased phospho-EGFR expression in the MUP-uPA background liver was attenuated by HNK treatment, HNK treatment clearly suppressed EGFR and FKBP5 protein expressions in the background NASH liver. Notably, EGFR protein levels were drastically reduced by HNK treatment (Figure 3B). EGFR suppression by HNK treatment led to the downregulation of ERK and mTORC1 signaling, the major downstream pro-tumorigenic pathways (Figure 3B). Notably, S6K and S6, which are the major mTORC1 targets, were downregulated by HNK treatment (Figure 3B).

### 2.4. HNK Induces MIG6 Expression Leading to EGFR Downregulation

The transcriptional regulation of EGFR expression is poorly understood [18,19], although epigenetic regulation such as DNA methylation and histone H3 modifications at the EGFR promoter has been reported. On the contrary, the EGFR degradation mechanism have been extensively studied. The ligand EGF promoted EGFR degradation via a negative feedback loop in human epithelial cells [20].

It has been reported that HNK can induce EGFR degradation by inhibiting heat shock protein 90 (HSP90), a molecular chaperone, in lung cancer cells, and in subcutaneously implanted mouse models [21]. Recently, it was reported that MIG6, which is encoded by *ERRFI1*, is an important inhibitor of EGFR signaling that is capable of inducing EGFR degradation [22]. Loss of MIG6 could account for the elevation of EGFR expression and signaling in several cancer types [23], including HCC [24]. MIG6 expression is induced by GR activation, which results in the translocation of GR from the cytoplasm to the nucleus [25]. Interestingly, FKBP5, which is also upregulated in the NASH-affected liver and suppressed by HNK in the present study, is a molecular chaperone that binds GR and inhibits its nuclear translocation [26].

Nonetheless, EGFR degradation in HCC has not yet been fully explored. Next, we decided to investigate whether HNK induces EGFR degradation. Interestingly, HNK treatment tended to upregulate *Errfi1* mRNA expression in HFD-fed MUP-uPA livers, in contrast to suppression of *Egfr* and *Fkbp5* mRNA expression (Figure 3A).

To confirm whether HNK also induces MIG6/*ERRFI1* expression and downregulates EGFR, we measured *ERRFI1* expression following HNK treatment in HCC cell lines (Figure S2). We decided to use Hep3B and Huh6 cells because HNK strongly induced *ERRFI1* expression in these two cell lines. To determine whether HNK downregulates EGFR through the GR–MIG6 axis, we firstly used Hep3B human HCC cells. HNK treatment inhibited the proliferation of HCC cells (Figure 3C) and induced *ERRFI1* mRNA expression in Hep3B cells (Figure 3D). HNK also reduced EGFR protein levels in parallel with MIG6 induction in a concentration-dependent manner (Figure 3E). Inhibition of EGFR expression was correlated with the downregulation of ERK and mTORC1 (Figure 3E). As observed in Hep3B cells, HNK treatment inhibited cell proliferation and reduced EGFR protein expression in parallel with MIG6 induction in Huh6 cells (Figure S3A–C). Inhibition of EGFR expression was correlated with the downregulation of ERK and mTORC1 (Figure S3C). When cells were stimulated with EGF, HNK treatment also downregulated ERK and mTORC1, and induced MIG6 more apparently (Figure S4).

### 2.5. MIG6 Knockout (KO) Abrogates the Inhibitory Effects of HNK on HCC Cell Proliferation

**Figure 4:**
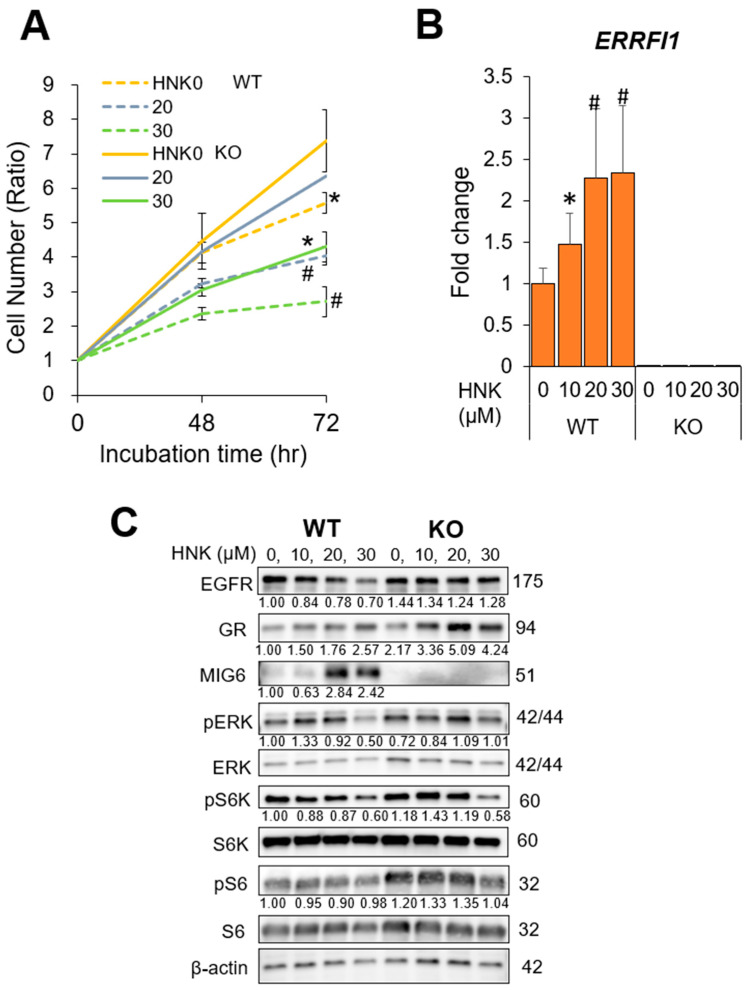
Mitogen-inducible gene 6 (MIG6) knockout (KO) abrogates the inhibitory effects of honokiol (HNK) on hepatocellular carcinoma (HCC) cell proliferation.(**A**) Viability and proliferation of parental and CRISPR MIG6 KO Hep3B cells 48 and 72 h after incubation with 0, 20, or 30 μM HNK. (**B**) Relative mRNA expression of *ERBB receptor feedback inhibitor 1* (*ERRFI1*) in HCC cells treated with 0, 10, 20, or 30 μM HNK (WT, wild-type Hep3B cells; KO, CRISPR MIG6-knockout clone cells). (**C**) Immunoblot analysis of parental and CRISPR MIG6 KO Hep3B cells 24 h after incubation with the indicated concentrations of HNK. Protein expression and phosphorylation of EGFR signaling-related molecules are presented with β-actin as a loading control. All graphs represent the mean ± SD (* *p* < 0.05, # *p* < 0.01 vs. the same cells without HNK treatment (HNK0, A) or vs. WT cells (HNK0, B)).

Next, we established MIG6/*ERRFI1*-knockout (KO) Hep3B and Huh6 cells using the clustered regularly interspaced short palindromic repeats (CRISPR)/Cas9 method to confirm the role of MIG6 in the effects of HNK on HCC cells. After confirming stable MIG6/*ERRFI1* deletion at both the mRNA and protein levels (Figure 4B,C, Figure S3B,C), we treated HCC cells with HNK. As expected, HNK inhibited EGFR expression and reduced ERK, S6K, and S6 phosphorylation in WT cells but not in KO cells (Figure 4C, Figure S3C). Accordingly, the KO cells grew more rapidly than parental cells regardless of HNK treatment (Figure 4A, Figure S3A), although high-concentration HNK (30 μM for Hep3B cells, 20 μM for Huh6 cells) partially suppressed cell proliferation (Figure 4A, Figure S3A).

These results strongly support the notion that HNK treatment inhibits cell proliferation by downregulating EGFR protein expression via MIG6/*ERRFI1* induction.

### 2.6. HNK Induces GR Nuclear Translocation, Leading to MIG6 Induction

**Figure 5:**
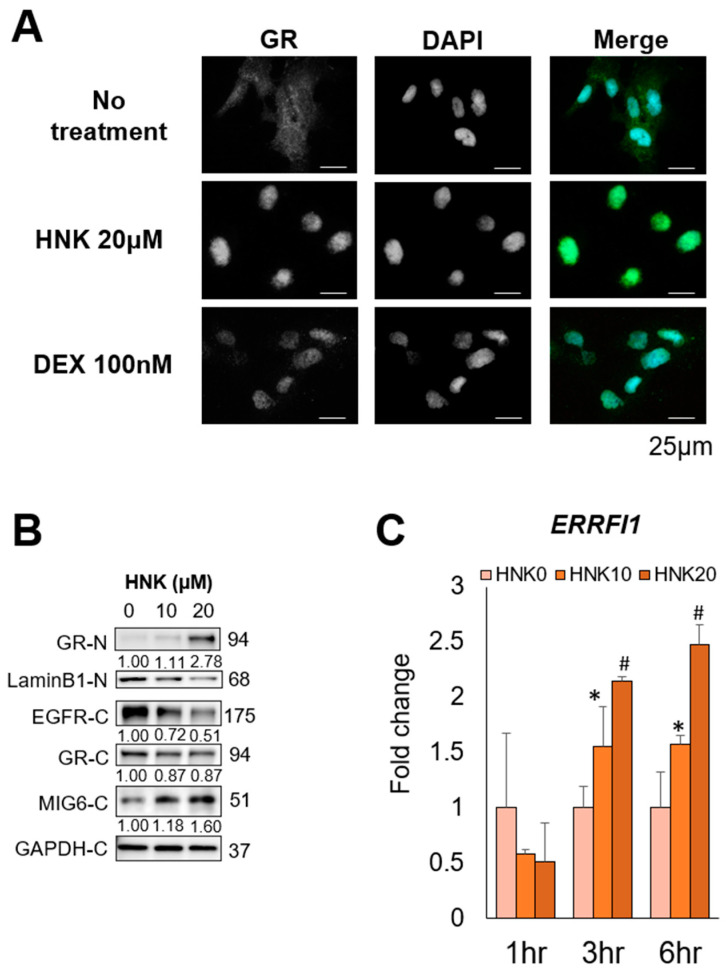
Honokiol (HNK) induces glucocorticoid receptor (GR) nuclear translocation, leading to mitogen-inducible gene 6 (MIG6) induction in Hep3B cells. (**A**) Subcellular localization of GR in Hep3B cells 2 h after incubation with the vehicle, 20 μM HNK, or 100 nM dexamethasone (DEX) was examined via immunofluorescence staining. 4′,6-Diamidino-2-phenylindole (DAPI) was used for nuclear counterstaining. (**B**) Immunoblot analysis of nuclear (-N) and cytoplasmic (-C) extracts from Hep3B cells 9 h after incubation with 0, 10, or 20 μM HNK. Protein expression of GR, epidermal growth factor receptor (EGFR), and MIG6 was examined. Lamin B1 (nuclear) and GAPDH (cytoplasmic) were used as loading controls. (**C**) Relative expression of *ERRFI1* mRNA in Hep3B cells treated with the indicated concentrations of HNK. All graphs represent the mean ± SD (* *p* < 0.05, # *p* < 0.01 vs. HNK0 condition at the same time course).

To determine where HNK induced MIG6 expression via GR activation, we examined its effects on GR expression and subcellular distribution. Although glucocorticoids are the classical GR ligands, glucocorticoid-independent modulation of GR activity has been described [27]. Indeed, we found that HNK treatment induced GR translocation to the nucleus in Hep3B cells, suggesting that HNK activated GR in a similar manner as the glucocorticoid dexamethasone (DEX) (Figure 5A,B). The same translocation was also observed in Huh6 cells (Figure S5A,B). In addition to GR activation, HNK treatment induced MIG6/*ERRFI1* expression. *ERRFI1* was rapidly induced by HNK, and this effect was accompanied by EGFR downregulation in both Hep3B (Figure 5B,C) and Huh6 cells (Figure S5B,C). By contrast, *EGFR* and *NR3C1* mRNAs levels remained unaltered for at least 6 h after HNK addition in these two cell lines (Figure S6A,B).

In addition, HNK treatment induced EGFR translocation into lysosome within the perinuclear compartment indicating that lysosomal degradation of EGFR was accelerated by HNK (Figure S7). Bafilomycin A, a lysosome inhibitor, enhanced the colocalization of EGFR with lysosome.

These results suggest that HNK degraded EGFR via activation of the GR–MIG6 axis.

### 2.7. GR Activation and ERRFI1 Expression Are Inversely Correlated with EGFR Expression in Human HCC

**Figure 6:**
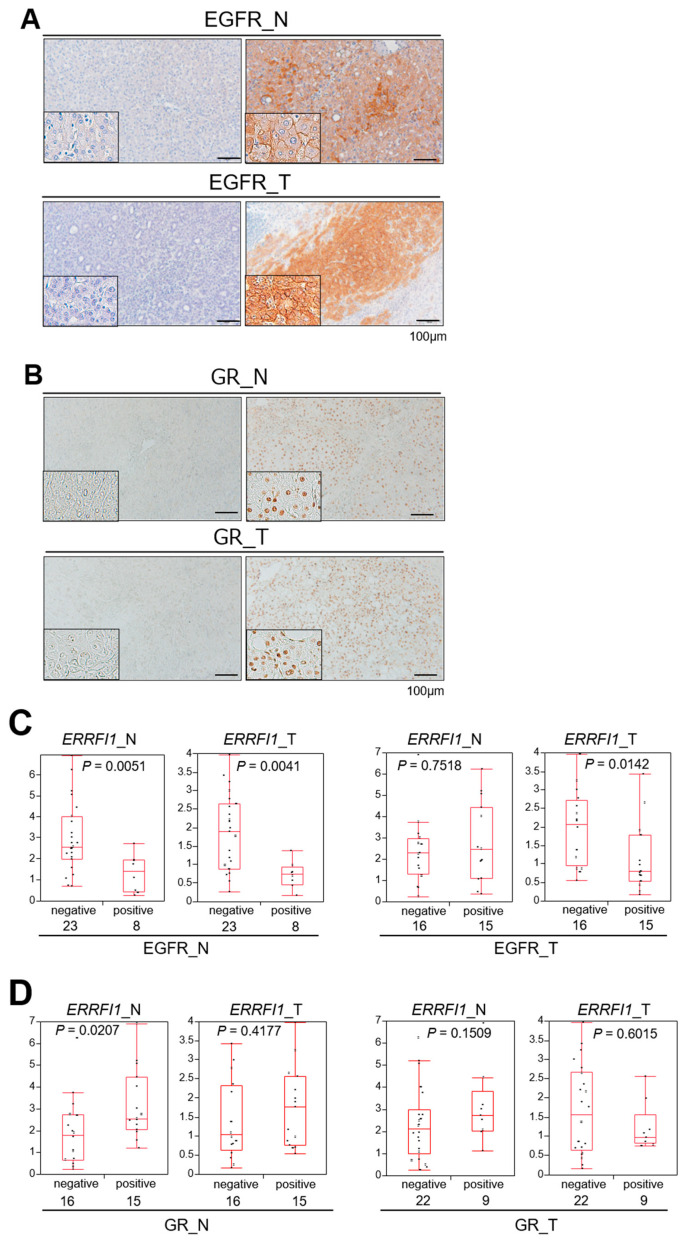
Glucocorticoid receptor (GR) activation and *ERBB receptor feedback inhibitor 1* (*ERRFI1*) expression are inversely correlated with epidermal growth factor receptor (EGFR) expression in human hepatocellular carcinoma (HCC). (**A**,**B**) Representative images of EGFR (A) and glucocorticoid receptor (GR; B) immunohistochemistry of non-tumor HCC adjacent tissue (N: left panels) and HCC (T: right panels). All samples were obtained via surgical resection in patients diagnosed with HCC. (**C**,**D**) Correlation between *ERRFI1* mRNA expression and positivity for EGFR (C) or GR (D) in non-tumor (N) and tumor (T) tissues. Each box plot depicts the median and quartiles. Whiskers indicate the furthest point within 1.5 × the interquartile range (the third quartile minus the first quartile) from the box.

The significance of the GR–MIG6 axis in the human HCC has not yet been reported. To confirm whether the GR–MIG6 axis modulates EGFR expression in human HCC, we performed GR and EGFR immunohistochemical staining in human HCC tissues and their corresponding non-tumor liver tissues (Table S2A,B). We also quantified *ERRFI1* expression by real-time qPCR. Immunohistochemical analysis illustrated that nearly half of the HCCs were EGFR-positive (15/31, 48.4%, Figure 6A,C) and that EGFR positivity of the cell membrane in non-tumor liver tissue was inversely correlated with nuclear GR positivity (*p* = 0.0373, Table S2C). EGFR-positive non-tumor tissues exhibited significantly lower *ERRFI1* expression than EGFR-positive HCC and non-tumor tissues (Figure 6A,C). In addition, EGFR-positive HCC had lower *ERRFI1* expression than EGFR-negative HCC (Figure 6C). These results suggested that decreased *ERRFI1* expression may increase EGFR expression during disease progression.

Non-tumor livers with GR-positive nuclei exhibited significantly higher *ERRFI1* expression, but this was not found in HCC tissues, supporting the notion that GR activation induces *ERRFI1* expression in non-tumor liver tissue (Figure 6B,D). In the human background liver, EGFR expression clearly depended on GR/*ERRFI1* expression coincident with the findings from experiments of HCC cells in vitro. As EGFR signaling is known as an oncogenic driver, we compared the background livers of EGFR- or GR-positive HCCs with those of EGFR- or GR-negative HCCs, and no significant difference was identified between the groups (Figure 6C,D).

### 2.8. ERRFI1 Expression Decreases in Parallel with NAFLD/NASH Progression

**Figure 7:**
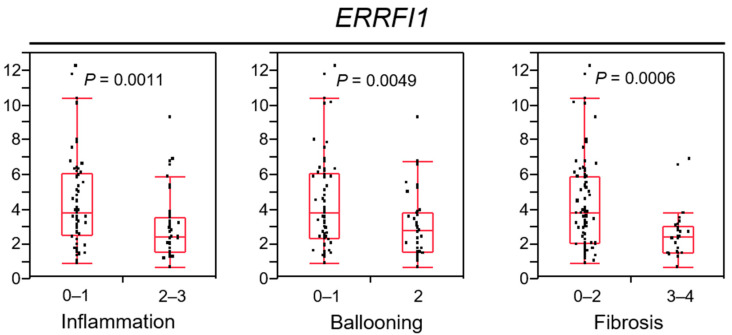
*ERBB receptor feedback inhibitor 1* (*ERRFI1*) expression decreases in parallel with non-alcoholic steatohepatitis (NASH) progression.

The GR–MIG6 axis appears important for the progression from chronic liver disease to HCC via EGFR suppression. Next, we evaluated *ERRFI1* expression in the livers of 105 patients with NAFLD to clarify the significance of its expression during NAFLD/NASH progression (Figure 7). The characteristics of the patients are described in Table S3. Focusing on liver histology, advanced NASH livers, which feature inflammation, ballooning and fibrosis, exhibited reduced *ERRFI1* expression.

These results suggested that *ERRFI1* levels decrease in parallel with NAFLD/NASH disease progression, and MIG6/*ERRFI1* suppression may contribute to HCC induction via EGFR degradation. To confirm the effect of HNK treatment on intracellular lipid accumulation, we incubated cells with 150 µM oleic acid, then stained with a fluorescent neutral lipid dye. HNK treatment clearly reduced the number of lipid droplets (Figure S8).

Correlations of *ERRFI1* mRNA expression with the inflammation grade (0–1, 2–3), ballooning grade (0–1, 2), and fibrosis stage (0–2, 3–4). Each box plot depicts the median and quartiles. Whiskers indicate the furthest point within 1.5 × the interquartile range (the third quartile minus the first quartile) from the box.

### 2.9. HNK Treatment also Attenuates a NAFLD-HCC Model

To confirm whether HNK treatment could suppress HCC development in another mouse model, we also tested its preventative effect in a DEN-induced HCC mouse model. As previously reported, HFD feeding accelerates HCC development and induces fatty liver, but not NASH, resembling obese people with NAFLD [28,29]. As a result, HNK treatment for the last 8 weeks of HCC development, i.e., 24–32 weeks of age, attenuated HCC development in DEN-treated mice fed an HFD (DEN-HFD mice, Figure S9A,B). The severity of liver steatosis did not differ between control and HNK treatment groups.

These results suggested that HNK exerts both direct anti-tumor and therapeutic effects on NASH/NAFLD. Notably, the anti-HCC effects of HNK were more apparent in HFD-fed MUP-uPA mice, the NASH-driven HCC model, than in DEN-HFD mice [10].

## 3. Discussion

EGFR is the third most studied gene/protein, being described in more than 40,000 papers, and it is often mutated in a variety of cancers [30]. However, the *EGFR* mutation rate in human HCC is low at approximately 1% [5,6]. Nonetheless, EGFR membrane expression has been observed in 40–70% of human HCCs [31,32,33], in line with our findings. Recently, it has reported that the HCC stem cell marker CD44 contributes to HCC initiation and progression through its interaction with EGFR in pericentral hepatocytes [34]. In this study, we observed that EGFR signaling is upregulated in the NASH liver and that the naturally occurring compound HNK prevents HCC induction by downregulating EGFR.

Several EGFR inhibitors were proven effective against epithelial-derived cancers, including lung, pancreatic, and colon cancers [4]. However, none of these inhibitors, including erlotinib, which exhibited anti-tumor activity and disease control in patients with unresectable or metastatic HCC in a phase 2 trial [35], have been approved for the treatment of advanced HCC because of the lack of survival benefits [7]. Nevertheless, a clinical study of EGFR inhibition using erlotinib in patients with cirrhosis evaluating its ability to inhibit the progression to HCC is ongoing (ClinicalTrials.gov: NCT02273362).

HCC usually arises in chronically inflamed livers, with inflammation being related to either viruses (HBV, HCV) or lifestyle factors (NASH, alcoholic liver disease). As EGFR signaling is important for liver regeneration [8,9], it is likely to be activated in the chronically inflamed liver. In concordance with these expectations, EGFR was overexpressed in the background liver of MUP-uPA mice. HNK may exert its inhibitory effects on EGFR and FKBP5 in the background liver and contribute to HCC prevention.

Recently, EGFR inhibition prevented the development of steatosis and liver injury in a mouse model of NAFLD [10,11]. This report also revealed that the effect of EGFR in the HFD-induced fatty liver phenotype was not shared by the receptor tyrosine kinase MET as investigated using MET KO mice. It is suggested that EGFR is a potential target for preventing NASH/NAFLD-derived HCC.

Assuming that EGFR drives NASH-to-HCC progression, and that HCC prevention requires the use of non-toxic agents, especially in patients with particularly low liver function, we focused on HCC development and tested the preventative ability of HNK, a naturally occurring compound known to have anti-inflammatory and anti-oxidant activities [14]. We found that HNK clearly inhibited NASH-induced HCC in the MUP-uPA mouse model. Although HNK attenuated NASH development, e.g., steatosis and fibrosis when administered to MUP-uPA mice, HNK administration more clearly suppressed hepatocarcinogenesis in MUP-uPA mice than DEN-HFD mice. These results suggest that HNK prevents both HCC but NASH development depending on the disease condition or stage, although the detailed mechanism of the effects of HNK on NASH development requires further investigations.

Mechanistically, HNK exerts its anti-tumorigenic activity through the activation of GR, which was found to accumulate in the nuclei of HNK-treated HCC cells. Once activated, GR induces MIG6 expression. MIG6, also known as Receptor-associated late transducer (RALT), shares significant homology with the protein product of rat gene-33 [23]. MIG6 is a cytoplasmic protein postulated to act as a tumor suppressor in lung, skin, breast, pancreatic, and ovarian cancers [23,36,37]. Indeed, whole-body *ERRFI1* deletion in mice led to the development of rectal, gastric, gall bladder, bile duct, lung, and skin tumors [23,36]. Although little is known about the role of MIG6 in liver cancer, *ERRFI1* deletion has been found in 13% of humans with HCC, in contrast to the extremely low incidence (around 1%) of *EGFR* mutations [38]. It was also reported that MIG6 is downregulated in human HCC, and this was correlated with increased EGFR expression [24]. That report also illustrated that MIG6 KO mice exhibited increased EGFR protein expression and enhanced hepatocyte proliferation after partial hepatectomy, supporting the notion that MIG6 is an endogenous inhibitor of EGFR signaling. Focusing on HCC, a previous study analyzed human specimens via immunohistochemistry (IHC) and identified low MIG6 expression and high cyclin D1 expression as independent predictors of reduced survival [39]. miR-589-5p is a potential prognostic marker of HCC that regulates tumor cell growth by targeting MIG-6, although the study did not examine EGFR or the GR–MIG6 axis [40]. Another report revealed that miR-374a could activate EGFR and AKT/extracellular signal-regulated kinase (ERK) signaling by regulating MIG-6 in HepG2 cells, but the mechanism was not pursued [41].

**Figure 8:**
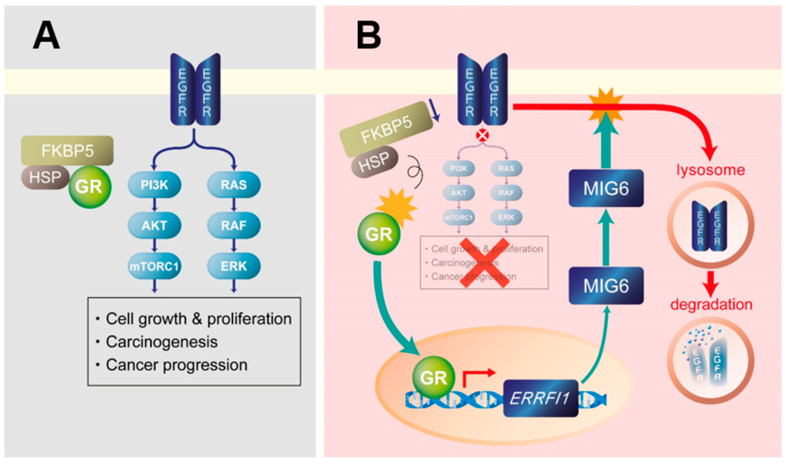
Proposed mechanism of glucocorticoid receptor (GR)–mitogen-inducible gene 6 (MIG6)-mediated epidermal growth factor receptor (EGFR) degradation and suppression by honokiol. (**A**) EGFR signaling plays a pivotal role in liver tumorigenesis by activating RAS/RAF/ERK and mechanistic (or mammalian) target of rapamycin complex 1 (mTORC1) signaling. FK506 binding protein 5 (FKBP5) and heat shock protein (HSP) cooperate to stabilize GR and inhibit its nuclear translocation in EGFR-expressing hepatocellular carcinoma cells. (**B**) Honokiol may cause GR to dissociate from the FKBP5/HSP complex. Once GR becomes free, it translocates to the nucleus and induces MIG6/*ERRFI1* transcription to eventually cause the lysosomal degradation of EGFR. As a result, EGFR signaling and its downstream targets RAS/RAF/ERK and mTORC1 are suppressed.

Notably, the molecular chaperone FKBP5 (also known as FKBP51 and FKBP54) is upregulated in HFD-fed MUP-uPA mice and is suppressed by HNK treatment. FKBP5 is a co-chaperone that maintains GR in an inactive state, together with other co-chaperones including HSP90 [26]. When the affinity of the GR complex is changed by FKBP5 displacement, GR translocates to the nucleus in an active state (Figure 8). Curiously, FKBP5 is also known to act as an oncoprotein through its ability to modulate GR activity and lead to alterations of target gene expression [17]. Collectively, these findings support the view that the GR–MIG6 axis is involved in EGFR degradation to suppress the progression of NASH to HCC.

HNK has exhibited anti-tumor effects in a variety of cancers, including breast, kidney, lung, pancreas, and liver cancers [42,43,44,45,46]. HNK has been reported as an anti-oxidant [14], and oxidative stress plays important roles in NASH pathophysiology [47], HNK may be involved in mouse liver and NASH-induced HCC by suppressing oxidative stress. However, little is known about its mechanisms of action, although previous studies implicated the activation of sirtuin 3 or LKB1 and the inhibition of β-catenin, NF-kappa B, or angiogenesis in the pharmacological effects of HNK [48,49]. Although the prevalence of systemic metastasis of HCC is not frequent and patients with HCC commonly die of the primary tumor burden and liver dysfunction, it was reported that HNK exerted suppressive effects in HCC metastasis model and in a xenograft model [15]. The same group also reported that HNK can induce EGFR degradation by inhibiting HSP90 in lung cancer cells and in subcutaneously implanted mouse models [21]. However, the ability of HNK to trigger EGFR degradation via the GR–MIG6 axis has not been reported, particularly in HCC. We found that HNK induced GR translocation to the nucleus, MIG6 expression, and subsequent EGFR degradation, and we propose that this pathway is responsible for the ability of HNK to interfere with NASH-to-HCC progression (Figure 8).

Although HNK degraded EGFR protein in HCC cells (Figure 5B and Figure S5B) without the increased of EGFR mRNA expression (Figure S6A,B), HNK suppressed *Egfr* and *Fkbp5* mRNA expression upregulated in HFD-fed MUP-uPA livers (Figure 3A), whose mechanism(s) should be further investigated. Since epigenetic changes and microRNAs involvement in NASH development have been reported [50,51], HNK may regulate the mRNA expression of *Egfr* and *Fkbp5* via epigenesis or microRNAs.

In human samples, we first revealed that EGFR-positive HCCs and their corresponding liver tissues displayed decreased *ERRFI1* expression. In addition, GR-positive non-tumor liver tissues featured lower EGFR expression and higher *ERRFI1* mRNA expression than GR-negative tissues. In addition, *ERRFI1* expression in NAFLD patients decreased in parallel with disease progression, i.e., severe inflammation, strong ballooning degeneration, and advanced fibrosis. Although only eight samples among our human HCC samples belonged to the non-B non-C group to which NASH-derived HCC supposedly belongs, the fact that patients with advanced NASH exhibited decreased *ERRFI1* expression and increased EGFR expression support the clinical importance of the MIG6–EGFR axis during NASH-induced HCC development. Therefore, the induction of *ERRFI1* expression in response to GR activation by HNK could be applicable to HCC prevention in patients with chronic liver diseases, especially NASH/NAFLD. Notably, some herbal drugs that are widely used in clinical practice in Japan and other Asian countries include HNK as a constituent. Although we clearly illustrated that HNK prevented the development of NASH-related HCC via the GR–MIG6–EGFR degradation pathway, further studies are needed to validate this disease prevention mechanism in humans.

In summary, we revealed that HNK suppresses the progression of NASH/NAFLD to HCC in mouse models. Our results suggest that HNK functions by inducing the nuclear translocation of GR and subsequent induction of MIG6, a negative regulator of EGFR protein expression. Our analysis of human HCC and NAFLD samples supported the clinical importance of the MIG6–EGFR axis during NASH-induced HCC development. EGFR has important roles in NAFLD/NASH progression and HCC development, and it can be inhibited by HNK via the aforementioned degradation mechanism. These findings could facilitate the use of other compounds that target the GR–MIG6–EGFR degradation pathway and exhibit the same preventative activity and efficacy as HNK.

## 4. Materials and Methods

### 4.1. Animals

As a result of sex differences in HCC prevalence, all mice used in this study were male. Mice were maintained in filter-topped cages under a 12-h–12-h dark–light cycle at KPUM and fed an autoclaved HFD (60% fat, 20% protein, and 20% carbohydrates based on caloric content; D12492, Research Diets, NJ, USA) and water. MUP-uPA mice were kindly provided by E.P. Sandgren (University of Wisconsin-Madison) and backcrossed with C57BL/6J mice for at least 12 generations [14]. Genotyping was performed, as previously described [14], and the sibling littermates served as controls. Hepatocytes in MUP-uPA newborn mice sustain damage as a result of ER stress induced by high uPA expression, which peaks at approximately 6 weeks of age. Inhibition of ER stress prevents both NASH and HCC development [13]. Importantly, similar to the characteristics of human HCC [15], the mutational spectrum of HCCs derived from MUP-uPA mice involves diverse activation of different oncogenic signaling pathways [5,6].

To generate mice with DEN-induced HCC and fatty livers, diethylnitrosamine (25 mg/kg, N0258, Sigma–Aldrich, St. Louis, MO, USA) was injected intraperitoneally into 14-day-old male mice [16,17]. After 4 weeks, the mice were fed an HFD until being sacrificed at 32 weeks of age. HNK was dissolved in phosphate-buffered saline (PBS) with Intralipid^®^ 20% and prepared at a concentration of 10 mg/kg immediately before injection into mice. The vehicle control consisted of PBS with Intralipid^®^ 20%. All the mice were divided randomly into two groups, the honokiol or vehicle control group.

### 4.2. Human Samples

Human HCC and adjacent non-tumor tissues were obtained from patients who underwent surgical resection at Saiseikai Suita Hospital from 2015 to 2018. Human NAFLD samples were collected from patients who underwent liver biopsy at KPUM from 2013 to 2019. NAFLD was diagnosed according to findings of steatosis in ≥5% of hepatocytes in liver biopsy specimens and the exclusion of other liver diseases, including viral hepatitis, autoimmune hepatitis, and drug-induced liver disease. Patients with a daily alcohol consumption >30 g for men or >20 g for women were excluded.

Liver tissues were fixed in 10% formalin, embedded in paraffin, sectioned, and either stained with hematoxylin and eosin (H&E) or processed for immunohistochemistry (IHC) using antibodies against EGFR and glucocorticoid receptor (GR). H&E staining and IHC were performed, as previously described [18] (details are described in the Supplementary Materials).

### 4.3. Liver Histology

The liver biopsy specimens were stained with H&E and Masson’s trichrome stain. The specimens were evaluated independently by two well-trained hepatologist at KPUM who were blinded to the clinical findings. An adequate liver biopsy sample was defined as that with a length >1.5 cm and more than 11 portal tracts. Steatosis affecting <5%, 5–33%, 33–66%, and >66% of the liver was assigned steatosis scores of 0, 1, 2, and 3, respectively. Lobular inflammation grades of 0, 1, 2, and 3 corresponded to none, mild, moderate, and severe, respectively. Ballooning scores of 0, 1, and 2 were classified as none, few, and many ballooned hepatocytes.

The NAFLD activity score was calculated as the sum of the steatosis, lobular inflammation, and hepatocellular ballooning scores. The severity of hepatic fibrosis was staged as follows: stage 1, zone 3 perisinusoidal fibrosis; stage 2, zone 3 perisinusoidal and portal fibrosis; stage 3, zone 3 perisinusoidal, portal, and bridging fibrosis; and stage 4, cirrhosis.

### 4.4. RNA Array Analysis

We used a Clariom S Assay microarray (Applied Biosystems/Thermo Fisher Scientific, Waltham, MA, USA) for the RNA array analysis of mouse liver samples. The results were analyzed using MicroArray Data Analysis Tool version 3.2. by Filgen, Inc. (Nagoya, Japan). 

### 4.5. RNA Isolation and Quantitative RT-PCR

We extracted and purified RNA using TRIzol^®^ (#15596018, Invitrogen/Thermo Fisher Scientific, Waltham, MA, USA) and chloroform and isopropanol, respectively. RNA (1 μg) was reverse-transcribed to generate cDNA using a PrimerScript RT cDNA Synthesis Kit (#RR036A, Takara Bio, Shiga, Japan). Individual gene expression was quantified by real-time qPCR using SYBR^®^ FAST qPCR Master Mix (#KK4602, KAPA BIOSYSTEMS, Wilmington, MA, USA) and a LightCycler 96 Real-Time PCR system (Roche Diagnostics, Mannheim, Germany). Gene expression was normalized to the expression of a housekeeping control gene (*GAPDH* or *GUSB*). The primers used for real-time qPCR analyses are listed in the Supplementary Materials.

### 4.6. Human HCC Cells

The HCC cell lines Hep3B (RRID:CVCL_0326) and Huh6 (CVCL_1296) were maintained according to the instructions provided by ATCC and the Japanese Collection of Research Bioresources Cell Bank (JCRB), respectively. Cell lines from ATCC and JCRB have been thoroughly tested and authenticated. All cells were obtained directly from ATCC or JCRB and passaged in our laboratory for fewer than 6 months after resuscitation. They were incubated with the indicated concentration of HNK for the specified period of time. The relative rates of cell viability and proliferation were determined by cell counting or using Cell Count Reagent SF (Nacalai Tesque, Kyoto, Japan). Nuclear extraction was performed using NE-PER^®^ Nuclear and Cytoplasmic Extraction Reagents (Thermo Fisher Scientific) according to the manufacturer’s instructions. To analyze GR translocation to the nucleus, Hep3B and Huh6 cells were incubated with serum-free Dulbecco’s Modified Eagle’s Medium (DMEM) for 6 h. Then, the medium was changed to serum-free DMEM supplemented with HNK (20 μM) or dexamethasone (DEX; 100 nM) and maintained for the indicated time.

### 4.7. Immunoblot Analysis

Liver samples and harvested HCC cells were homogenized in RIPA buffer, and then equal amounts of liver homogenates were fractionated via SDS-PAGE and transferred onto a polyvinylidene fluoride membrane. The membrane was incubated with antibodies against EGFR, phospho-EGFR (tyrosine 992), FK506 binding protein 5 (FKBP5), mitogen-inducible gene 6 (MIG6), ERK, phospho-ERK, p70S6K, phospho-p70S6K (Thr389), S6, phospho-S6, GAPDH, lamin B1 (all from Cell Signaling Technology, Danvers, MA, USA), GR (#24050-1-AP, Proteintech Group, Rosemont, IL, USA), and β-actin (A1978, Sigma–Aldrich, St. Louis, MO, USA). The antibodies used for immunoblotting are listed in the Supplementary Materials. Densitometric analysis of blots were performed, and the ratio of phosphorylated–total protein or total protein–loading control were indicated.

### 4.8. Immunofluorescence Analysis

Immunofluorescence analysis was performed for Hep3B and Huh6 cells treated with HNK or DEX, as previously described [18]. Briefly, cells were incubated on a glass chamber slide with the indicated drug, covered, and incubated with ice-cold 100% methanol for 10 min at −20 °C. After washing with PBS, cells were blocked with diluted donkey serum for 30 min at room temperature. Anti-GR (#24050-1-AP, Proteintech Group, Rosemont, IL, USA) and Alexa Fluor 488-conjugated donkey anti-rabbit IgG (#11-545-152, Jackson ImmunoResearch Inc., West Grove, PA, USA) antibodies were used as the primary and secondary antibodies, respectively. After washing with PBS, slides were mounted with medium containing DAPI (VECTASHIELD H-1500, Vector Laboratories, Burlingame, CA, USA). A BZ-X700 fluorescence microscope (Keyence Corporation, Osaka, Japan) was used to assess the expression and subcellular localization of GR, EGFR, LAMP1, and lipids.

### 4.9. Clonal Line Generation of MIG6/ERBB Receptor Feedback Inhibitor 1 (ERRFI1)-Knockout (KO) HCC Cells

Genome editing experiments involving ribonucleoprotein (RNP) lipofection in HCC cells were performed using the Alt-R CRISPR-Cas9 system (Integrated DNA Technologies, IA, USA) according to the manufacturer’s instructions. Briefly, crRNA (Hs.Cas9.ERRFI1.1.AC) and tracrRNA (CRISPR-Cas9 tracrRNA) were mixed at an equimolar ratio to produce guide RNA oligos. Then, Cas9 (S.p. HiFi Cas9 Nuclease V3) and Opti-MEM were added to form RNP, which was transfected into HCC cells using Lipofectamine CRISPRMAX transfection reagent (Thermo Fisher Scientific). Additional details of this experiment are described in the Supplementary Materials.

### 4.10. Statistical Analysis

Data are presented as the mean ± SD or as the median with interquartile ranges, as indicated. Differences in medians, means, and two categorical variables were analyzed using Wilcoxon’s signed-rank test (JMP8.0, SAS Institute Inc., Cary, NC, USA), Student’s t-test, or Fisher’s exact test (GraphPad Prism-6, GraphPad Software, La Jolla, CA, USA), respectively. *p* < 0.05 was considered significant.

## 5. Conclusions

We revealed that HNK suppresses the progression of NASH/NAFLD to HCC in mouse models by inducing the nuclear translocation of GR and subsequent induction of MIG6, a negative regulator of EGFR protein expression. Our analysis of human HCC and NAFLD samples supported the clinical importance of the MIG6–EGFR axis during NASH-induced HCC development.

## Data Availability

The raw data for RNA array in this study have been deposited in NCBIs Gene Expression Omnibus (GEO, http://www.ncbi.nlm.nih.gov/geo/) and are accessible through GEO Series accession number GSE163692.

## Supplementary Materials

The following are available online at https://www.mdpi.com/2072-6694/13/7/1515/s1. Figure S1: HNK treatment decreases body weight gain and attenuates liver injury in MUP-uPA mice, Figure S2: Honokiol induces *ERRFI1* induction in HCC cells, Figure S3: MIG6 knockout abrogates the inhibitory effects of HNK on Huh6 cell proliferation, Figure S4: Honokiol treatment suppresses EGFR signaling, Figure S5: HNK induces glucocorticoid receptor (GR) nuclear translocation, leading to mitogen-inducible gene 6 (MIG6) induction in Huh6 cells, Figure S6: Honokiol does not rapidly induce *EGFR* or *NR3C1* mRNA expression, Figure S7: EGFR translocates into lysosome and accumulated within perinuclear compartment upon HNK exposure, Figure S8: Honokiol treatment attenuates lipid accumulation, Figure S9: Honokiol treatment attenuates HCC development in DEN-HFD mice, Table S1. Differentially expressed genes between HFD-fed MUP-uPA and HFD-fed wild type mouse livers, Table S2. GR activation and *ERRFI1* expression are inversely correlated with EGFR expression in human HCC, Table S3. Characteristics of 105 patients with non-alcoholic fatty liver disease (NAFLD), File 1. Uncropped western blots figures
