# Supplementary material for: Honokiol Prevents Non-Alcoholic Steatohepatitis-Induced Liver Cancer via EGFR Degradation through the Glucocorticoid Receptor—MIG6 Axis
Source: Cancers (Basel). 2021 Mar 25;13(7):1515. doi: 10.3390/cancers13071515 (PMC8037653; doi:10.3390/cancers13071515)
Supplement: Supplementary file 1 [file cancers-13-01515-s001.zip › supplementary final/file 1 Uncropped western blots figures.pdf]

# Fig3B

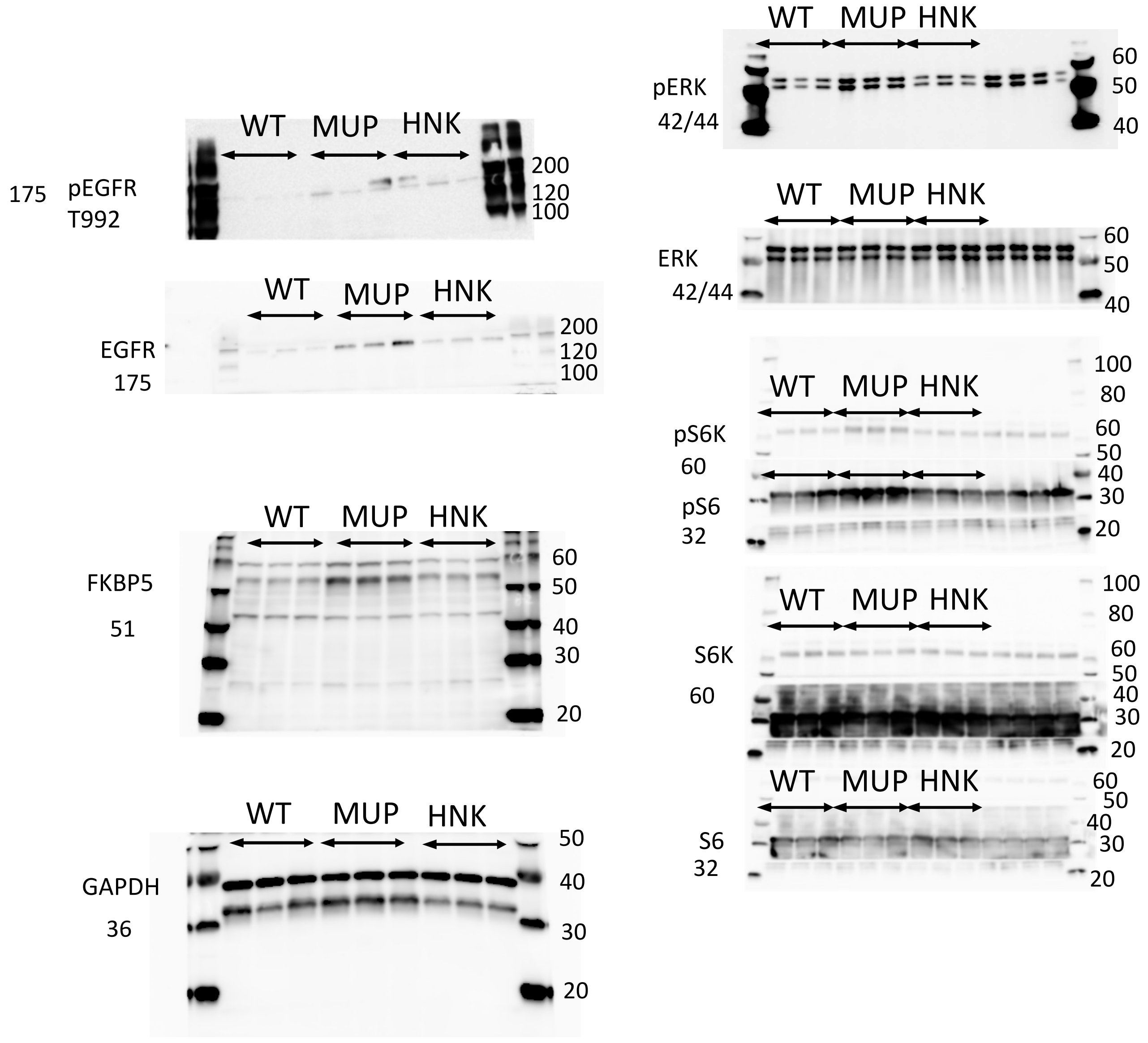


The antibodies used for immunoblotting are listed in the Supplementary Materials and Methods.

# Fig3E

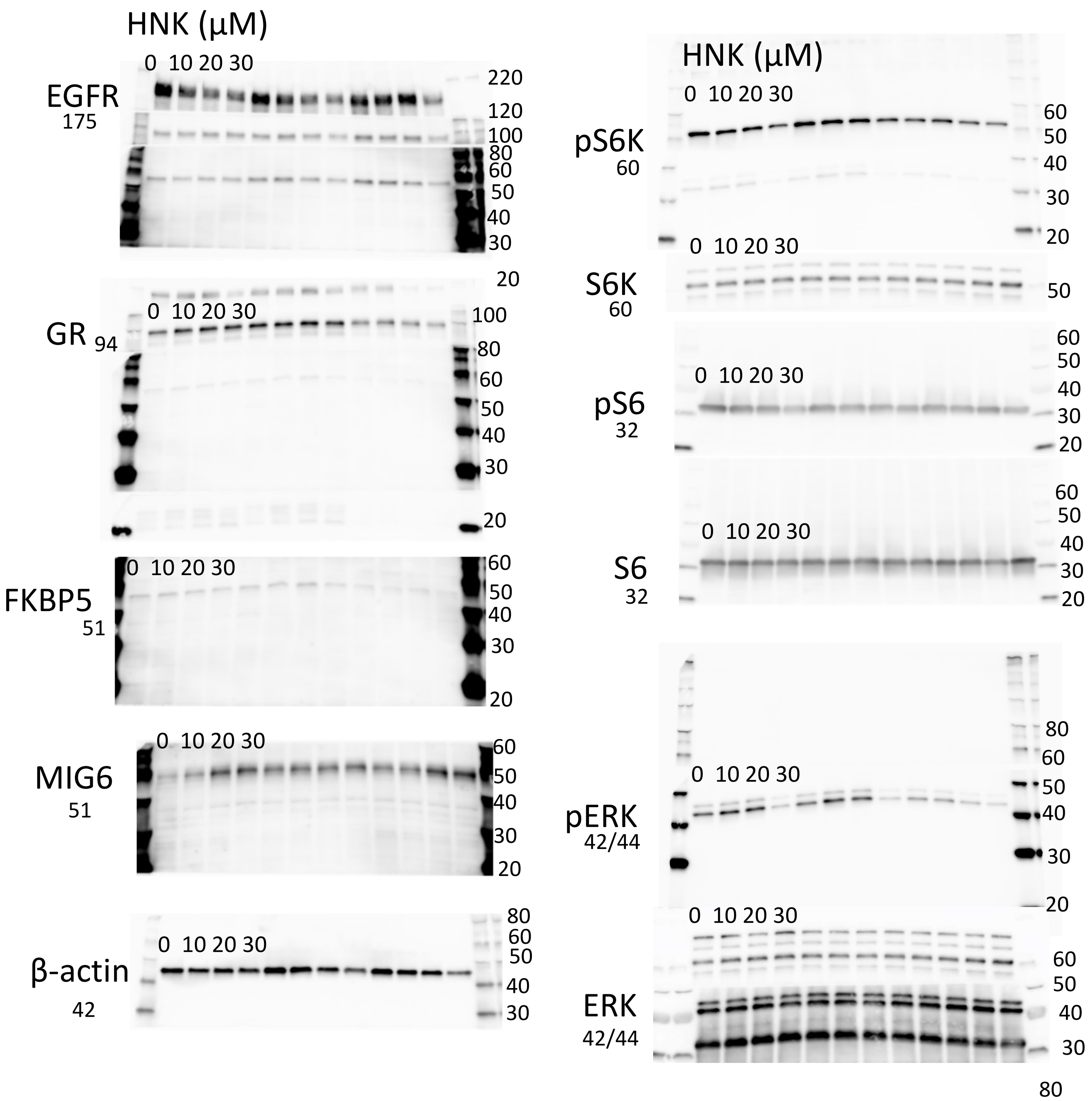
HNK (μM)
0 10 20 30
EGFR
175
220
120
100
80
60
50
40
30
GR 94
20
100
80
60
50
40
30
20
FKBP5
51
MIG6
51
β-actin
42
pS6K
60
S6K
60
pS6
32
S6
32
pERK
42/44
ERK
42/44

# Fig4C

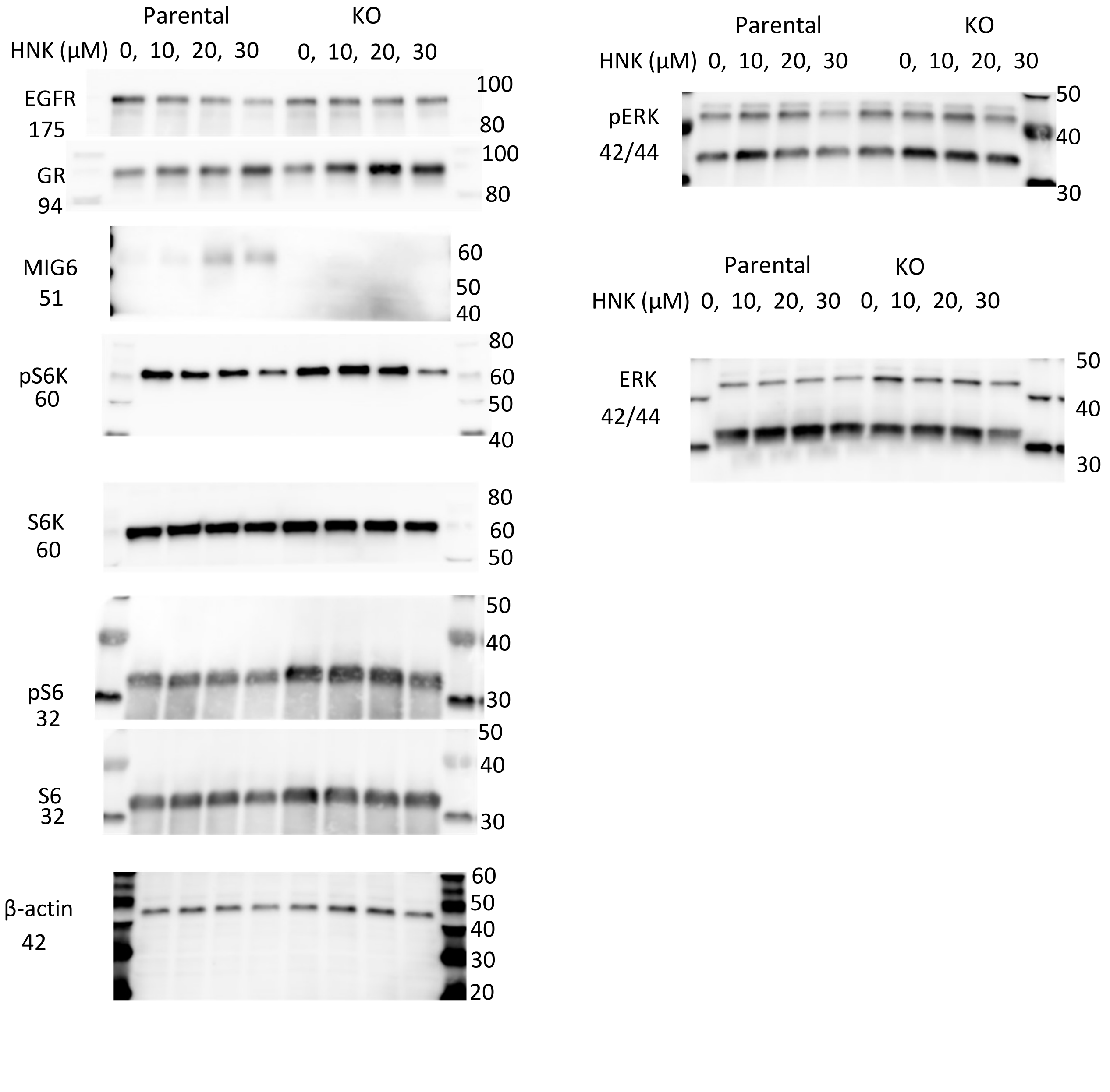
Parental
KO
HNK (μM) 0, 10, 20, 30 0, 10, 20, 30
EGFR
175
100
80
GR
94
100
80
MIG6
51
60
50
40
pS6K
60
80
60
50
40
S6K
60
80
60
50
pS6
32
50
40
30
S6
32
50
40
30
β-actin
42
60
50
40
30
20
Parental
KO
HNK (μM) 0, 10, 20, 30 0, 10, 20, 30
pERK
42/44
50
40
30
Parental
KO
HNK (μM) 0, 10, 20, 30 0, 10, 20, 30
ERK
42/44
50
40
30

# Fig5B

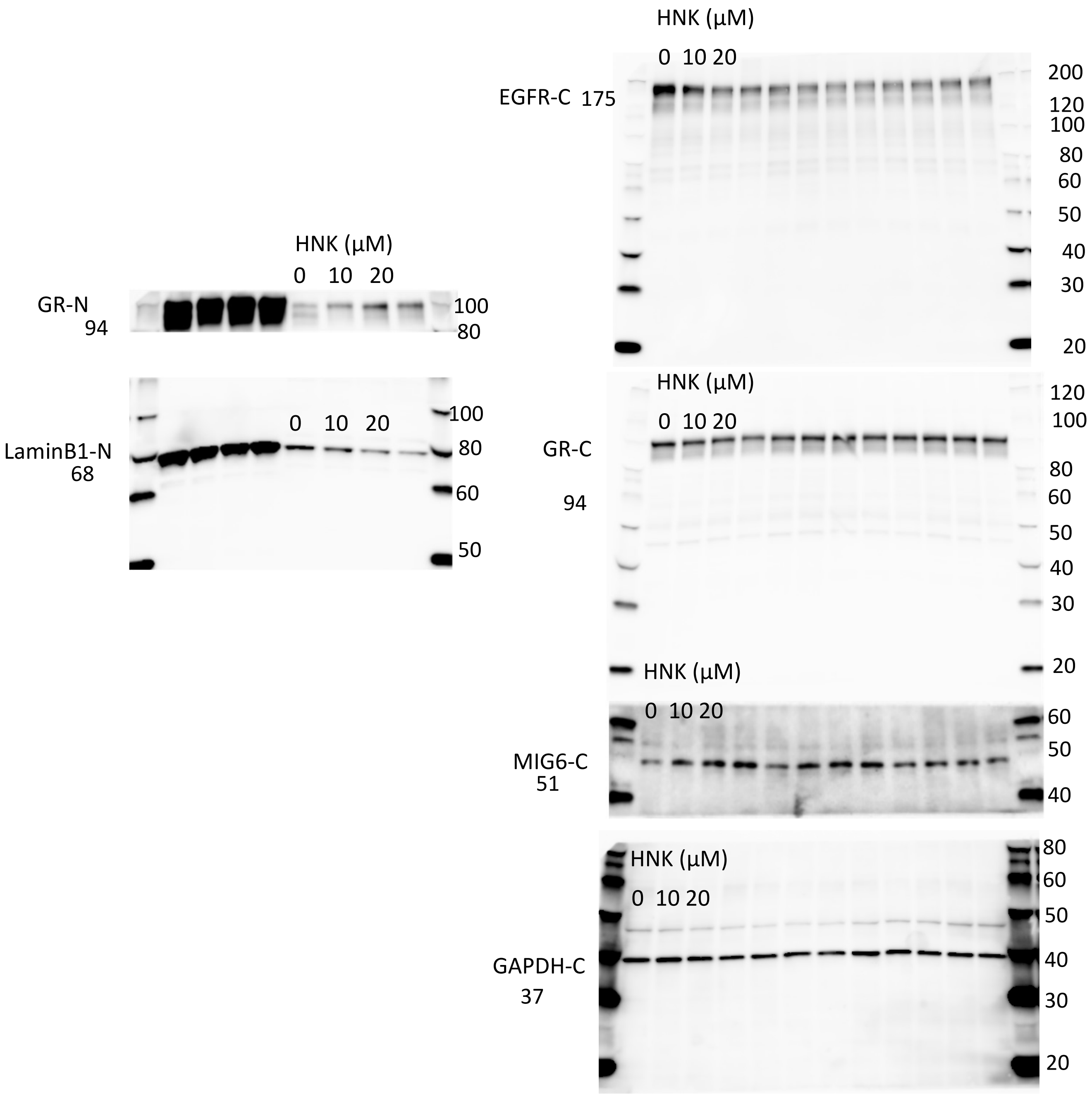

HNK (µM)
0 10 20
EGFR-C 175
200
120
100
80
60
50
40
30
20
HNK (µM)
0 10 20
GR-N
94
100
80
LaminB1-N
68
100
80
60
50
HNK (µM)
0 10 20
GR-C
94
120
100
80
60
50
40
30
20
HNK (µM)
0 10 20
MIG6-C
51
60
50
40
HNK (µM)
0 10 20
GAPDH-C
37
80
60
50
40
30
20

# Figure S3C

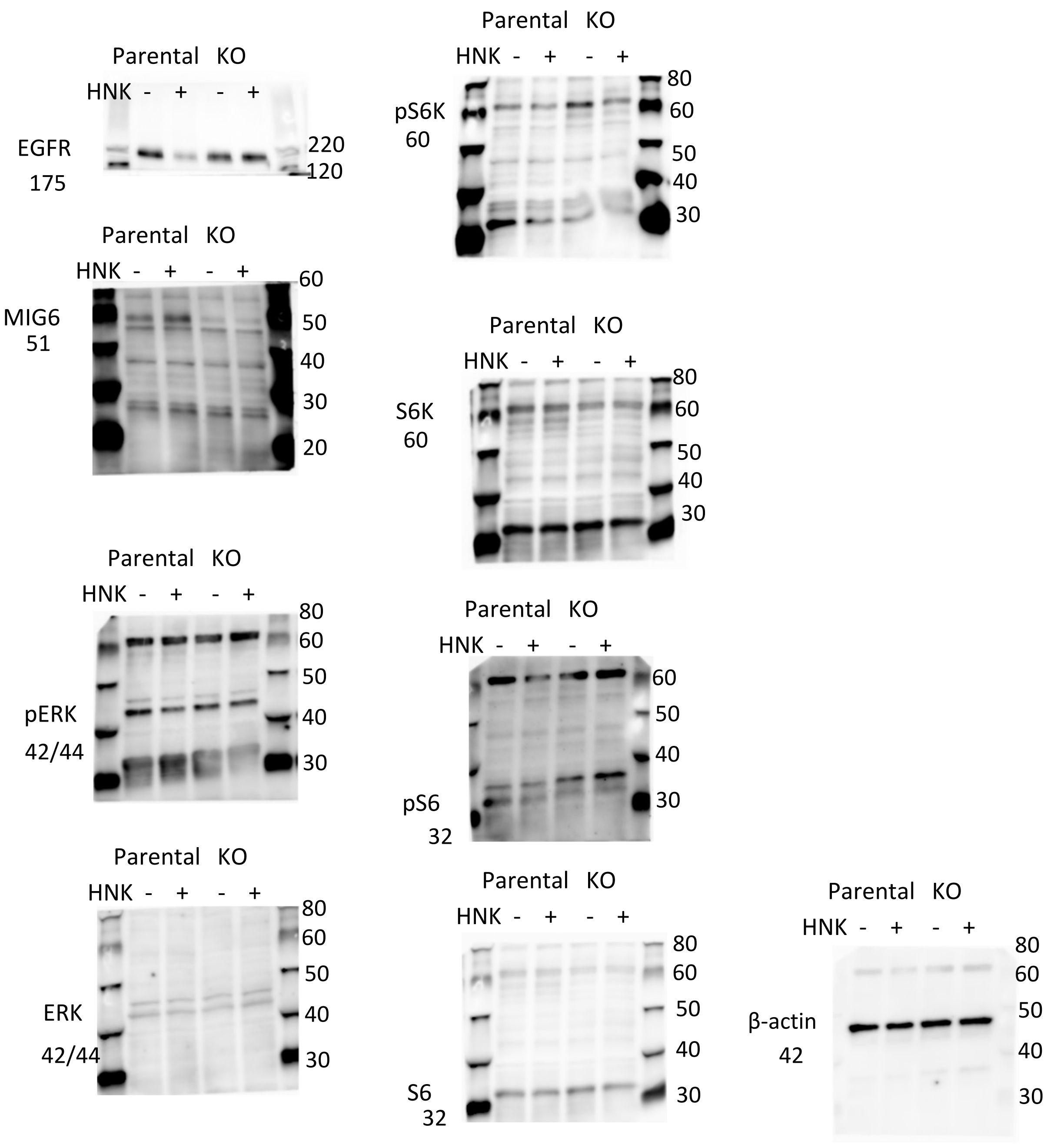
Parental KO
HNK - + - +
EGFR
175
220
120
MIG6
51
60
50
40
30
20
pERK
42/44
80
60
50
40
30
ERK
42/44
pS6K
60
S6K
60
pS6
32
S6
32
β-actin
42

# Figure S4A-Hep3B

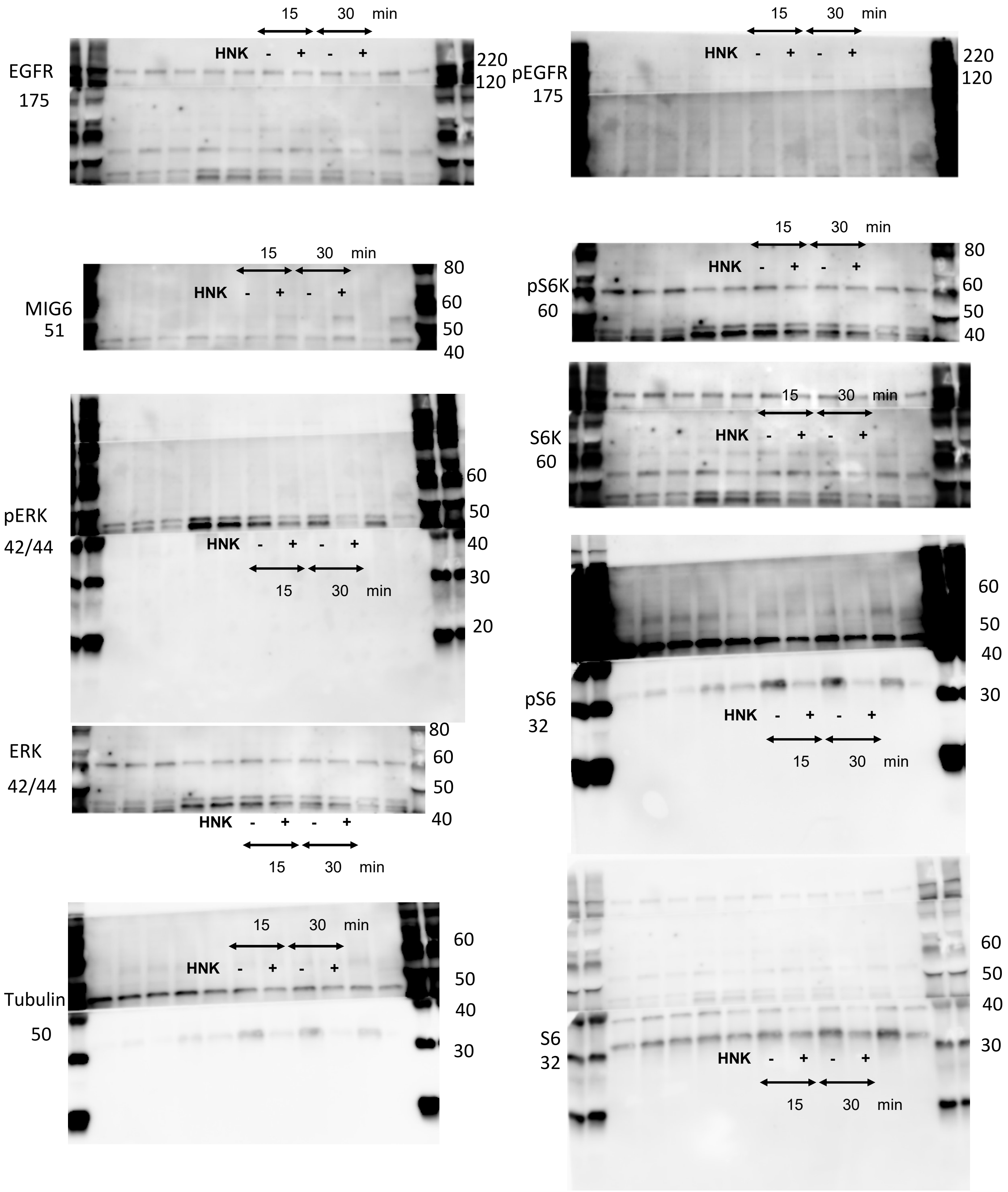

# Figure S4B-Huh6

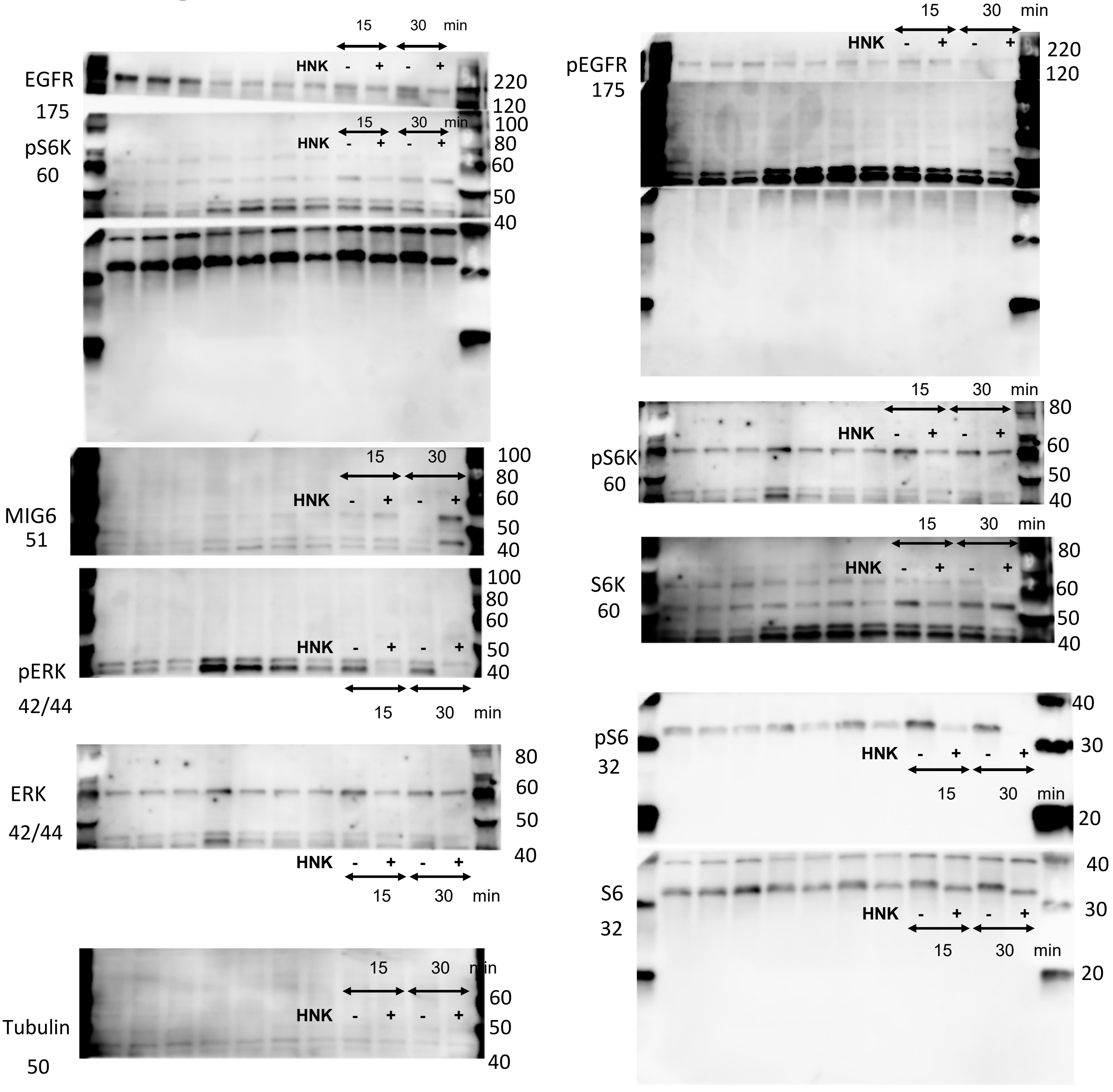

# Figure S5B

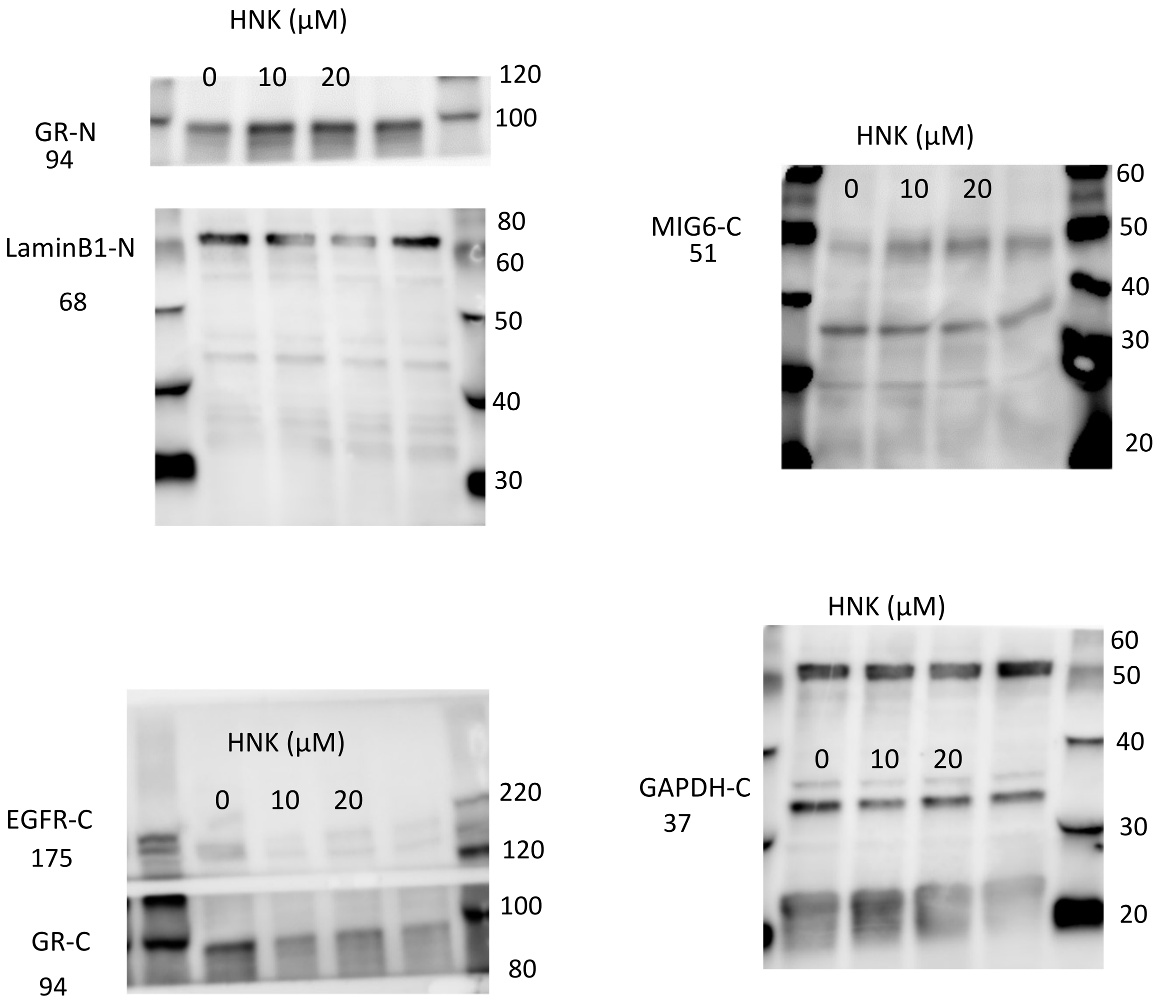
HNK (µM)
0
10
20
120
100
GR-N
94
80
60
50
40
30
LaminB1-N
68
HNK (µM)
0
10
20
60
50
40
30
20
MIG6-C
51
HNK (µM)
0
10
20
220
120
100
80
EGFR-C
175
GR-C
94
HNK (µM)
60
50
40
30
20
0
10
20
GAPDH-C
37
