# Supplementary material for: Honokiol Prevents Non-Alcoholic Steatohepatitis-Induced Liver Cancer via EGFR Degradation through the Glucocorticoid Receptor—MIG6 Axis
Source: Cancers (Basel). 2021 Mar 25;13(7):1515. doi: 10.3390/cancers13071515 (PMC8037653; doi:10.3390/cancers13071515)
Supplement: Supplementary file 1 [file cancers-13-01515-s001.zip › supplementary final/Supplementary material.docx]

Article

Honokiol Prevents Non-Alcoholic Steatohepatitis-Induced Liver Cancer via EGFR Degradation through the
Glucocorticoid Receptor—MIG6 Axis

**Supplementary Materials and Methods**

**Animals and liver analysis**

After mice were sacrificed, their livers were removed and separated into individual lobes. Externally visible tumors (>1 mm) were counted and measured. Large lobes were fixed in 10% formalin for 24–48 hours or embedded in Tissue-Tek OCT compound (Sakura Finetek) for paraffin or frozen block preparation, respectively. Paraffin-embedded liver tissues were used for H&E and Sirius red staining. Sirius red staining (sirius red F3B, #365548, Sigma-Aldrich) was performed to quantitate the amount of collagen fibers. Frozen tissue sections were stained with Oil red O (ORO, #O1391, Sigma-Aldrich) for lipid detection. Sirius Red-, Oil red O-positive areas were counted in 6–10 random fields (×100 or ×200) on each slide (1). All the quantification results are depicted in the bar graphs. Remaining lobes were micro-dissected into tumor and nontumor tissue and stored at ‐80 °C until analyzed.

**Immunohistochemistry (IHC) and immunofluorescent (IF) analysis**

IHC analysis was performed as described previously (2). Briefly, after human liver slides were deparaffinized, antigen retrieval was performed for 30 min with protease solution at room temperature (RT) for anti-EGFR antibody (#423701, Nichirei Biosciences, Tokyo, Japan) or citrate buffer at 96 °C for anti-GR antibody (#24050-1-AP, Proteintech Group, IL, USA). After cooling and washing with PBS, the slides were incubated with hydrogen peroxide at RT for 10min to block endogenous peroxidase activity. After washing with PBS, the slides were incubated with blocking solution (#X0909, DAKO/Agilent, CA, USA) for 30 min, then incubated at 4 °C overnight with antibodies diluted in the antibody diluent buffer (#S0809, DAKO/Agilent, CA, USA). After washing with PBS, the slides were incubated with corresponding secondary antibodies with HRP-labeled polymer for 1 hour followed by PBS washes, then developed with DAB chromogen for 2 min at RT. For EGFR staining, counterstaining with hematoxylin and rinse in running tap water for 5 min were performed. After dehydration, clearing and mounting were performed, the slides were extensively examined by BX-50 microscopy (Olympus Corporation, Tokyo, Japan). Oleic acid (#O3008, Sigma-Aldrich), BODIPY (#D3922, Thermofisher), anti-LAMP1 antibody (#sc-19992, Santa-Cruz) were used for IF analysis.

# List of primers used for quantitative RT-PCR

| **Species** | **Target Gene** | **Sequence (5' - 3')** | |
| --- | --- | --- | --- |
| human | *EGFR* | forward | AGGCACGAGTAACAAGCTCAC |
| human | *EGFR* | reverse | ATGAGGACATAACCAGCCACC |
| human | *FKBP5* | forward | AATGGTGAGGAAACGCCGATG |
| human | *FKBP5* | reverse | TCGAGGGAATTTTAGGGAGACT |
| human | *NR3C1* | forward | ACAGCATCCCTTTCTCAACAG |
| human | *NR3C1* | reverse | AGATCCTTGGCACCTATTCCAAT |
| human | *ERRFI1* | forward | CTGGAGCAGTCGCAGTGA |
| human | *ERRFI1* | reverse | GCCATTCATCGGAGCAGATTTG |
| human | *ERRFI1* (for CRISPR knockout cells) | forward | GACCCACCGAAGATTAAGAAGG |
| human | *ERRFI1* (for CRISPR knockout cells) | reverse | GGTCTAGGAGGTATGGGAACTCT |
| human | *GAPDH* | forward | TGACAACTTTGGTATCGTGGAAGG |
| human | *GAPDH* | reverse | AGGCAGGGATGATGTTCTGGAGAG |
| human | *GUSB* | forward | GTCTGCGGCATTTTGTCGG |
| human | *GUSB* | reverse | CACACGATGGCATAGGAATGG |
| mouse | *Egfr* | forward | GCCATCTGGGCCAAAGATACC |
| mouse | *Egfr* | reverse | GTCTTCGCATGAATAGGCCAAT |
| mouse | *Fkbp5* | forward | TGAGGGCACCAGTAACAATGG |
| mouse | *Fkbp5* | reverse | CAACATCCCTTTGTAGTGGACAT |
| mouse | *Nr3c1* | forward | AGCTCCCCCTGGTAGAGAC |
| mouse | *Nr3c1* | reverse | GGTGAAGACGCAGAAACCTTG |
| mouse | *Errfi1* | forward | TGGCCTACAATCTGAACTCCC |
| mouse | *Errfi1* | reverse | GACCACACTCTGCAAAGAAGT |
| mouse | *Gapdh* | forward | AACTTTGGCATTGTGGAAGG |
| mouse | *Gapdh* | reverse | CCCTGTTGCTGTAGCCGTAT |
| mouse | *Gusb* | forward | GGGTCAGTGTGTTGTTGATGG |
| mouse | *Gusb* | reverse | GCAGTTGTGTGGGTGAATGG |

**Human HCC cells**

Hep3B and Huh6 cells were incubated with honokiol as indicated concentration and time course. For assays with Cell Count Reagent SF, the cells were seeded in 96-well plates. After 12hours, the cells were washed with PBS and the medium was changed to either 100 μL of DMEM with control solution or HNK. After 24, 36, or 48 hour incubation, 10μL of WST-8 (a water-soluble tetrazolium dye) solution was added to the wells, then incubated for another 2 hours. The absorbance at 450 nm was measured to calculate relative cell viabilities.

**Clonal line generation of MIG6/ERBB receptor feedback inhibitor 1 (ERRFI1)-knockout (KO) HCC cells**

Twenty-four hours after transfection, medium was replaced with fresh one. Mutation detection was performed with T7 Endonuclease I using the Alt-R Genome Editing Detection Kit (Integrated DNA Technologies, IA, USA) according to the manufacturer’s instructions. Clonal cell lines were established from the bulk cells by manual colony picking. For manual colony picking, transfected cells were seeded at clonal density (250 cells per 35 mm dish) and incubated in the complete media for around 14 days for colony formation. From each dish, we picked 50 colonies manually with a P20 pipette. Colonies were later plated into 96-well plates and scaled up to larger dishes gradually. At 35 mm dish, cells were harvested and analyzed for successful knockout by immunoblot analysis with anti-MIG6 antibody. Among established clones, AC2E5 clone cells were used for further analysis.

**IDT Alt-R CRISPR-Cas9 system**

| Alt-R CRISPR-Cas9 crRNA, 2 nmol, Hs.Cas9.ERRFI1.1.AC |
| --- |
| Alt-R Cas9 HPRT Positive Ctrl crRNA Human, 2 nmol |
| Alt-R Genome Editing Detection Kit, 25 rxn |
| Alt-R S.p. HiFi Cas9 Nuclease V3, 100 µg |
| Alt-R CRISPR-Cas9 tracrRNA, 5 nmol |
| Alt-R HPRT PCR Primer Mix, Human, 2 nmol |
| Alt-R Cas9 Negative Control crRNA #1, 2 nmol |
| Alt-R CRISPR-Cas9 Control Kit, Human, 2 nmol |

**Antibodies for immunoblotting**

| Target | Catalog# | Company |
| --- | --- | --- |
| pEGFR tyr992 | 2235 | Cell signaling technology |
| EGFR | 4267 | Cell signaling technology |
| FKBP5 | 12210 | Cell signaling technology |
| GR | 24050-1-AP | Proteintech Group |
| MIG6 | 2440 | Cell signaling technology |
| pERK | 9101 | Cell signaling technology |
| ERK | 4695 | Cell signaling technology |
| S6K | 9202 | Cell signaling technology |
| pS6K Thr389 | 9205 | Cell signaling technology |
| pS6 | 4858 | Cell signaling technology |
| S6 | 2217 | Cell signaling technology |
| GAPDH | 5174 | Cell signaling technology |
| β-actin | A1978 | Sigma-Aldrich |
| laminB1 | 12586 | Cell signaling technology |
| Anti-rabbit IgG, HRP-linked | 7074 | Cell signaling technology |
| Anti-mouse IgG, HRP-linked | 7076 | Cell signaling technology |

**Supplementary Figures and Tables**


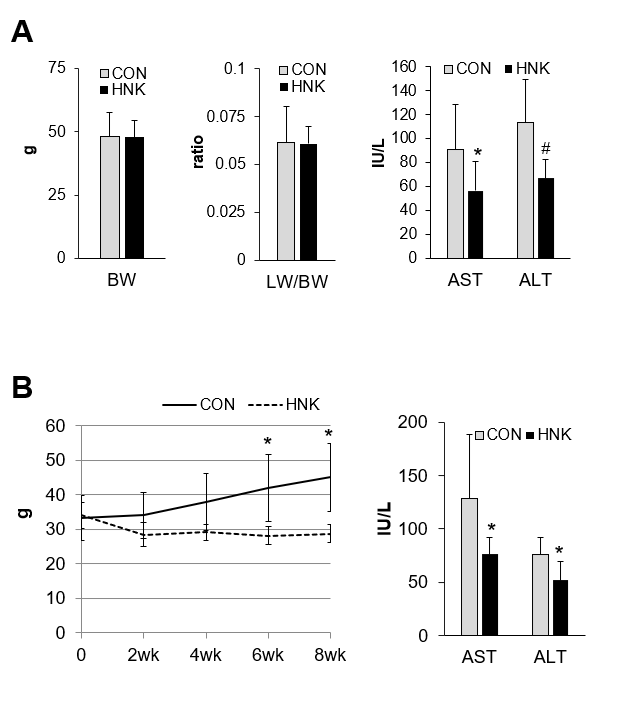


**Figure S1.** HNK treatment decreases body weight gain and attenuates liver injury in MUP-uPA mice. (A) Body weight (BW), liver weight / body weight ratio (LW / BW), serum AST and ALT levels of the HFD-fed MUP-uPA mice at 40 weeks of age after 8 weeks treatment with HNK or control. (B) Body weight change (left) and serum AST/ALT levels (right) of the HFD-fed MUP-uPA mice during HNK treatment from 12 to 20 weeks of age. All graphs represent means +/- SD (* *p* < 0.05, # *p* < 0.01).


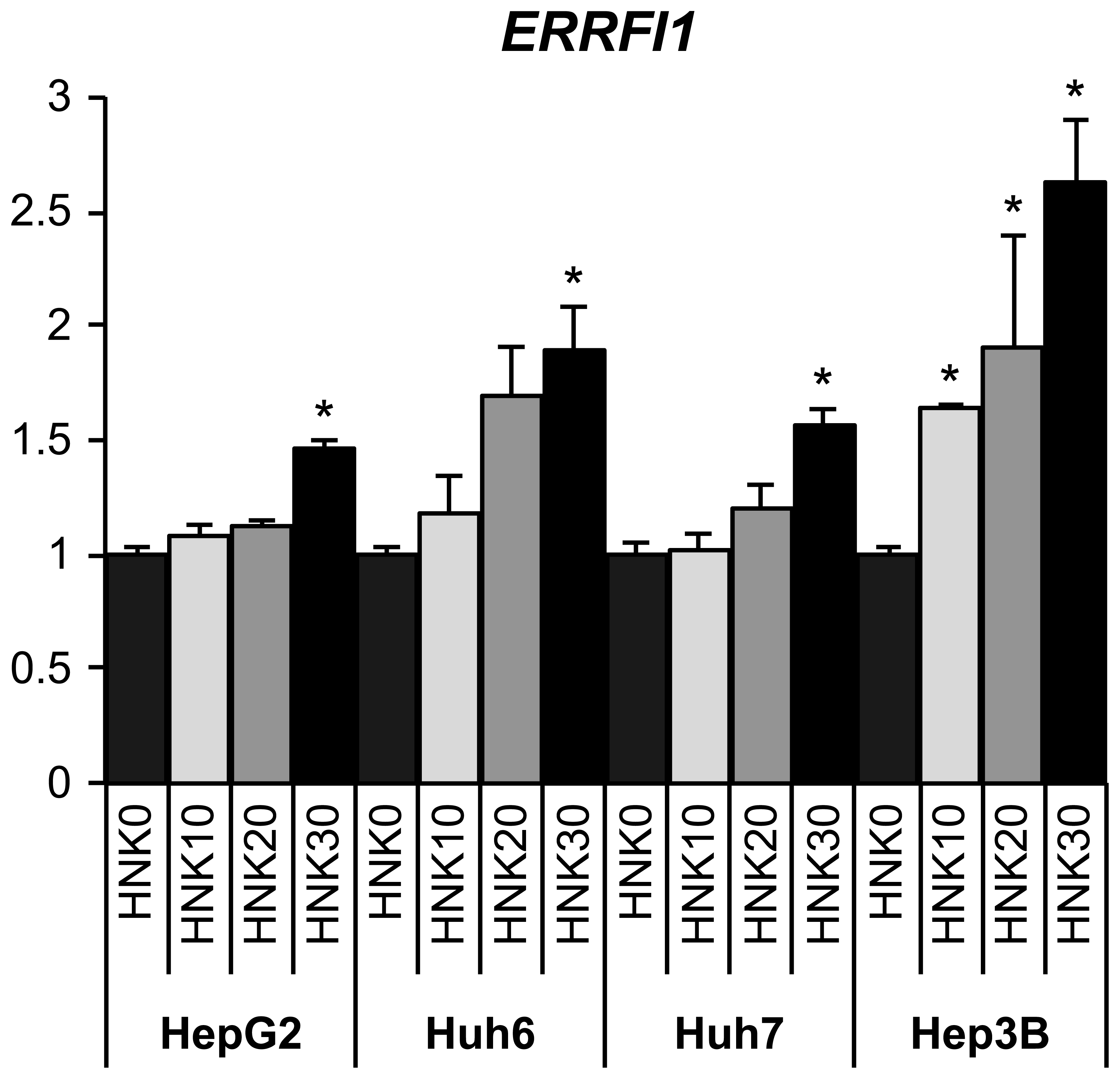


**Figure S2.** Honokiol induces ERRFI1 induction in HCC cells Relative expression of ERRFI1 in HCC cell lines 24 hours after incubation with 0, 10, 20, or 30 μM HNK. All graphs represent means +/- SD (* *p* < 0.05 vs the same cells without HNK treatment (HNK0)).


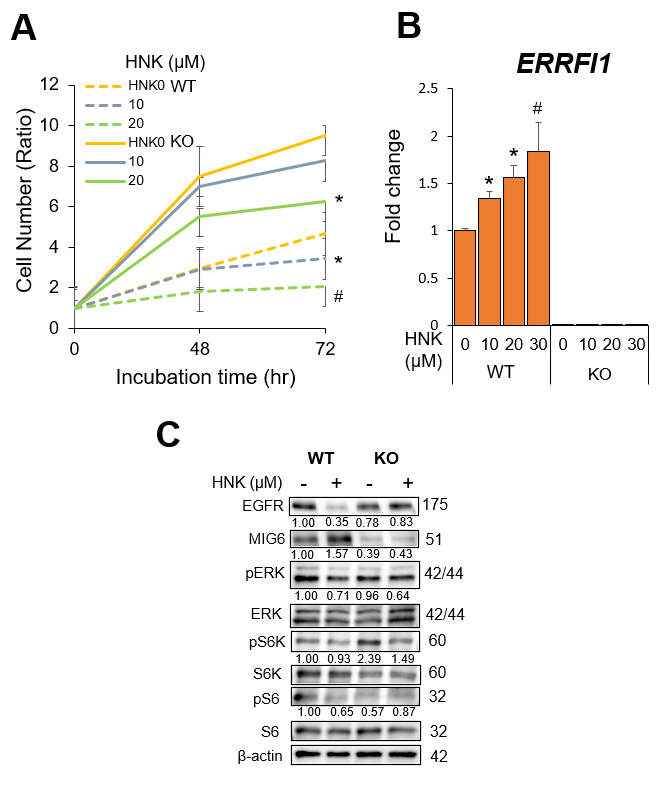


**Figure S3.** MIG6 knockout abrogates the inhibitory effects of HNK on Huh6 cell proliferation. (A) Cell viability/proliferation of parental and CRISPR MIG6 knockout Huh6 cells 48 and 72 hours after incubation with 0, 10, or 20 μM HNK. (B) Relative expression of ERRFI1 mRNAs in Hep3B cells treated with 0, 10, 20, or 30 μM HNK (WT, wild-type Huh6 HCC cells; KO, CRISPR MIG6 knockout clone cells). (C) Immunoblot analysis of parental and CRISPR MIG6 knockout HCC cells 24 hours after incubation with the indicated concentrations of HNK. Protein expression and phosphorylation of EGFR signaling-related molecules are shown with β-actin as a loading control. All graphs represent means +/- SD (* *p* < 0.05, # *p* < 0.01 vs the same cells without HNK treatment (HNK0) (A), or vs parental cells (HNK0) (B)).


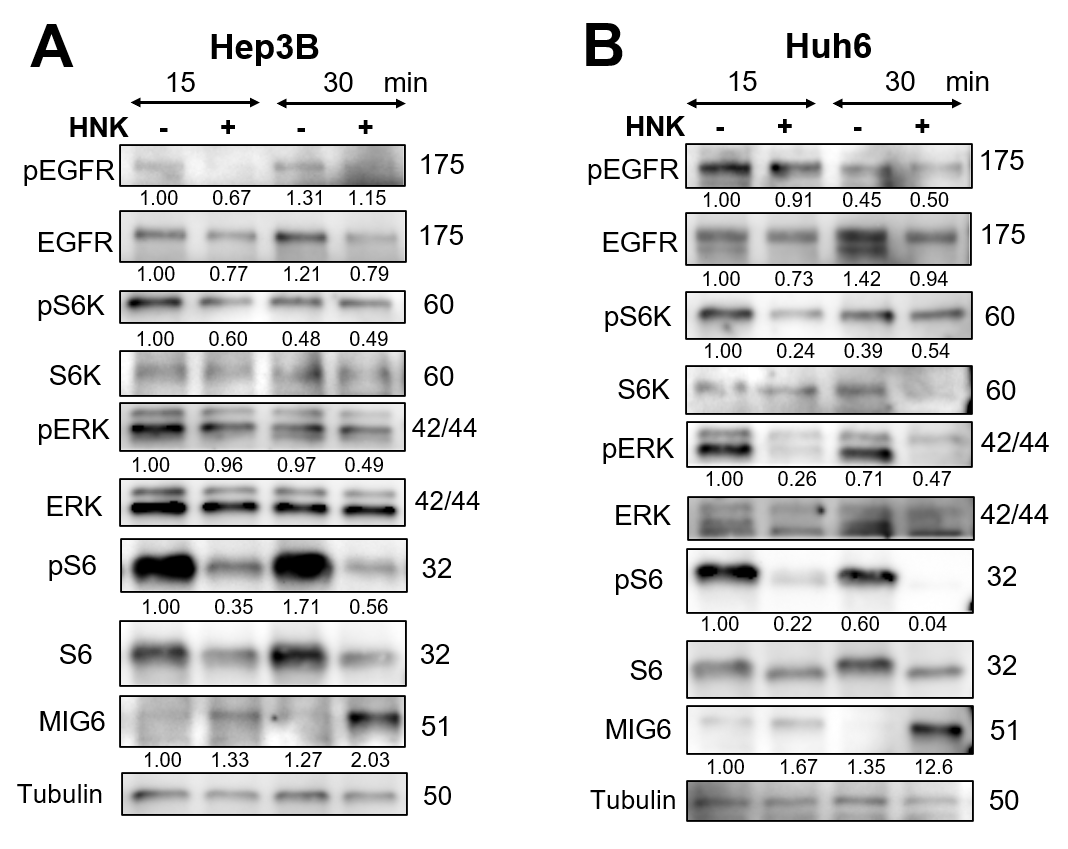
**Figure S4.** Honokiol treatment suppresses EGFR signaling. Immunoblot analysis of Hep3B (A) and Huh6 (B) cells 15 or 30 minutes after 50 ng/ml EGF stimulation, and incubation with either vehicle control or 20 μM HNK. Protein expression and phosphorylation of EGFR signaling-related molecules are presented. Tubulin was used as a loading control.


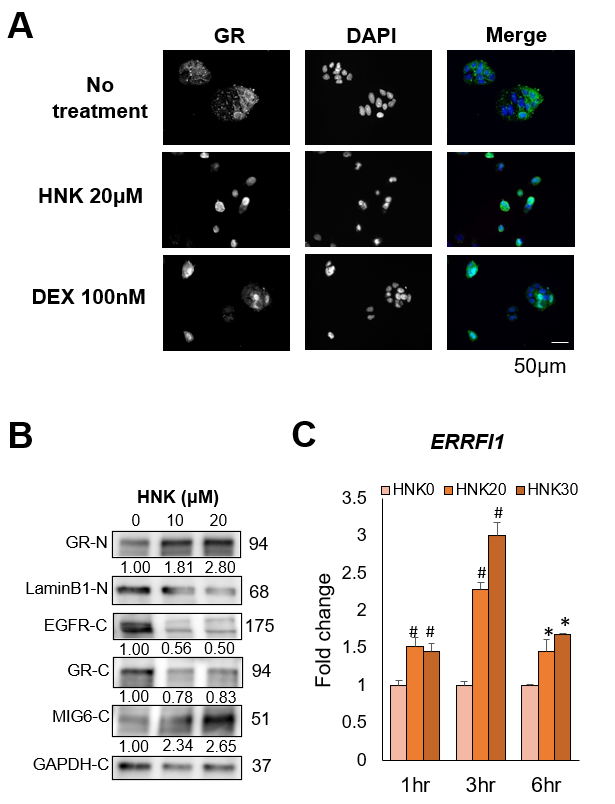


**Figure S5.** HNK induces glucocorticoid receptor (GR) nuclear translocation, leading to mitogen-inducible gene 6 (MIG6) induction in Huh6 cells. (A) Subcellular localization of GR in Huh6 cells 2 hours after incubation with vehicle control, 20 μM HNK, or 100 nM dexamethasone (DEX) was examined by immunofluorescence staining. DAPI (4′,6-diamidino-2-phenylindole) was used for nuclear counterstaining. (B) Immunoblot analysis of nuclear (N) and cytoplasmic (-C) extracts from Huh6 cells 9 hours after incubation with 0, 10, or 20 μM HNK. Protein expression of GR, EGFR and MIG6 was examined. Lamin B1 (Nuclear) and GAPDH (Cytoplasmic) were used as loading controls. (C) Relative expression of ERRFI1 mRNA in Huh6 cells treated with the indicated concentrations of HNK. All graphs represent means +/- SD (* *p* < 0.05, # *p* < 0.01 vs HNK0 condition at the same time course).


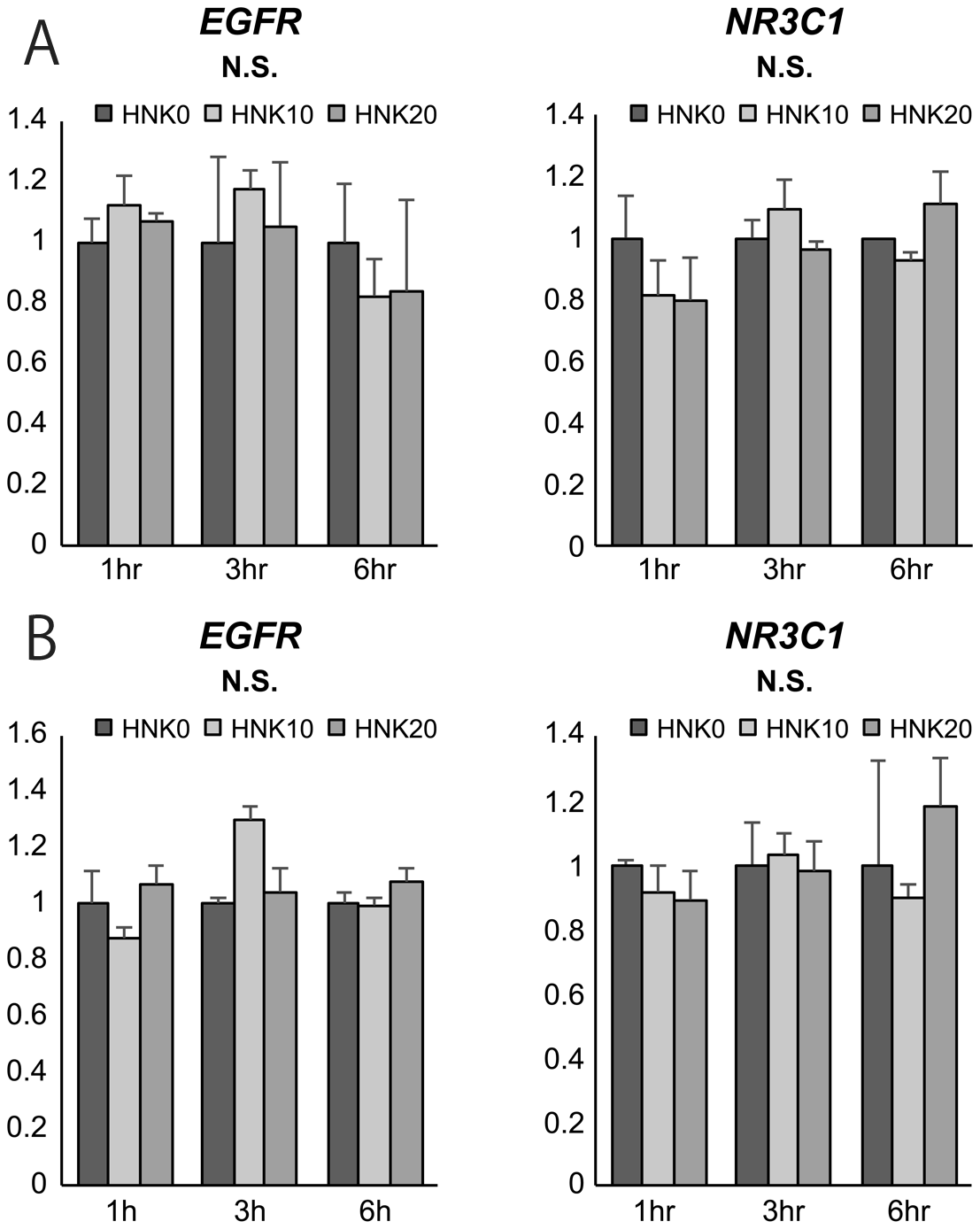


**Figure S6.** Honokiol does not rapidly induce *EGFR* or *NR3C1* mRNA expression. Relative expression of *EGFR* and *NR3C1* mRNAs 1, 3, and 6 hours after incubation with 0, 10, or 20 μM HNK in (A) Hep3B cells and (B) Huh6 cells (*n* = 3-4 per group, N.S.; not significant vs HNK0 condition at the same time course). All the graphs represent means +/- SD.


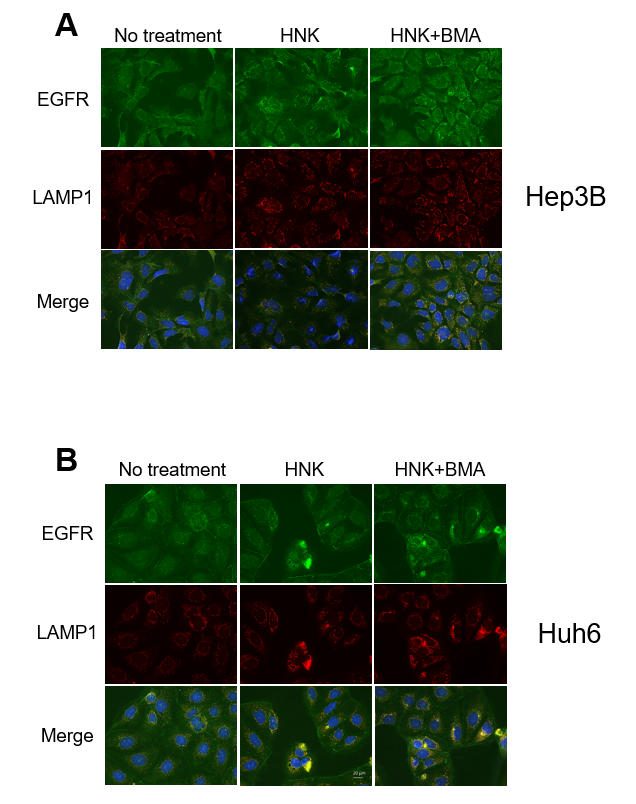


**Figure 7.** EGFR translocates into lysosome and accumulated within perinuclear compartment upon HNK exposure. Subcellular localization of EGFR and LAMP1 in Hep3B (A) and Huh6 (B) cells 6 hours after stimulation with 50 ng/ml EGF, and incubation with either vehicle control or 20 μM HNK. Then, cells were stained with anti-EGFR, anti-LAMP1, and DAPI. Bafilomycin A1 (BMA) impaired lysosomal function leading to lysosome accumulation within perinuclear compartment.


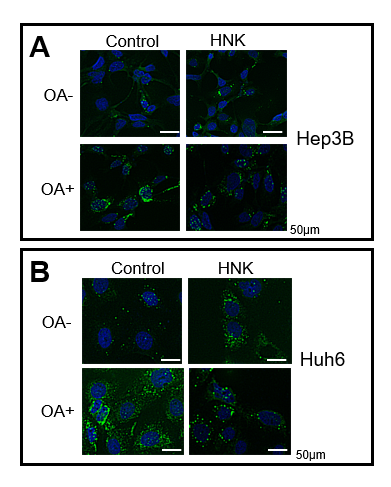


**Figure S8.** Honokiol treatment attenuates lipid accumulation. Lipid accumulation in Hep3B (A) and Huh6 (B) cells 6 hours after incubation with BSA control (OA-) or oleic acid (OA+) was assessed by stained with a fluorescent neutral lipid dye BODIPY (green). DAPI was used for nuclear counterstaining.


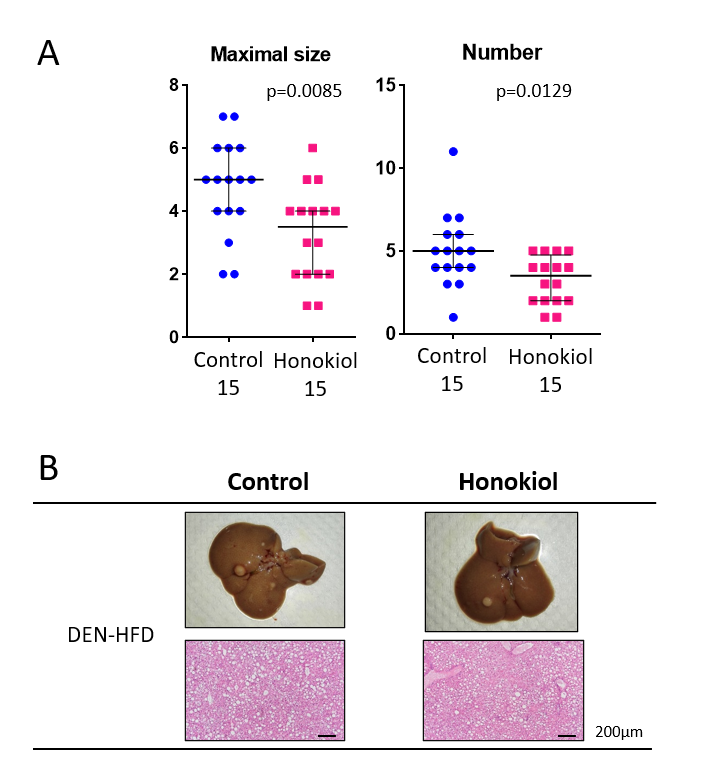


**Figure S9.** Honokiol treatment attenuates HCC development in DEN-HFD mice. (A) Maximal tumor size and tumor numbers in the diethylnitrosamine (DEN)-high fat diet (HFD) model treated with vehicle control or honokiol. For the DEN-HFD model, two weeks old males were injected with 25 mg/kg DEN and fed HFD from 6 weeks until 32 weeks of age when analyzed. Honokiol was injected into DEN-HFD mice for the last 8 weeks of HCC development, i.e. between 24-32 weeks of age (n = 8 per group). Tumor development was analyzed 2–4 days after the final honokiol injection. Results are presented as the median with interquartile ranges. (B) Gross morphology of livers with HCCs and typical histology of the background livers in mice of DEN-HFD (lower panels) model.

**Table S1. Differentially expressed genes between HFD-fed MUP-uPA and HFD-fed wild type mouse livers.** The list of the individual genes of Hierarchical clustering representation. Total 306 genes were differentially expressed between HFD-fed MUP-uPA (MUP) and HFD-fed wild type B6 (WT) mouse livers (*n* = 3 per group). Among them, 288 genes including *Egfr* and *Fkbp5* were upregulated ≥2.0 in MUP mouse livers compared to WT mouse livers (Please refer to the “Supp_TableS1” file).

**Table S2.** GR activation and *ERRFI1* expression are inversely correlated with EGFR expression in human HCC. (A) Characteristics of 31 patients with HCC analyzed in this study (missing data: AFP *n* = 2, PIVKA-2 *n* = 3). Data are indicated as median (minimum - maximum). (B) Etiology of chronic liver diseases with HCC: HBV, hepatitis B virus; HCV, hepatitis C virus; ALD, alcoholic liver disease; NBNC, non-B non-C virus infection (NASH patients are included in this category). (C) Correlation between GR and EGFR positivity in non-tumor HCC adjacent tissue (NT).


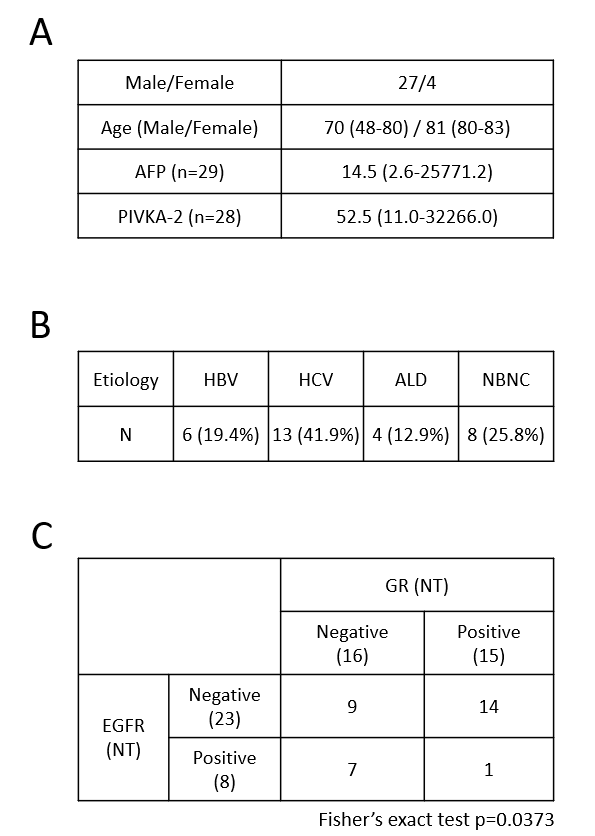


**Table S3. Characteristics of 105 patients with non-alcoholic fatty liver disease (NAFLD).** Abbreviations: BMI, body mass index; NAS, NAFLD activity score; ERRFI1, ERBB receptor feedback inhibitor 1.


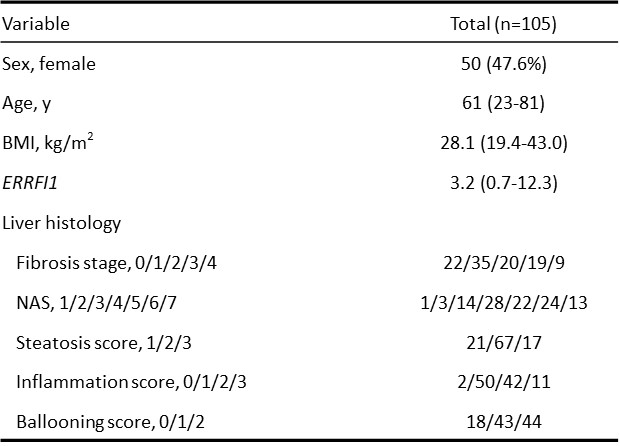


**Supplementary References**

1. Umemura A, Park EJ, Taniguchi K, Lee JH, Shalapour S, Valasek MA*, et al.* Liver damage, inflammation, and enhanced tumorigenesis after persistent mTORC1 inhibition. Cell Metab **2014**;20:133–144

2. Umemura A, He F, Taniguchi K, Nakagawa H, Yamachika S, Font-Burgada J*, et al.* p62, Upregulated during Preneoplasia, Induces Hepatocellular Carcinogenesis by Maintaining Survival of Stressed HCC-Initiating Cells. Cancer Cell **2016**;29:935–948
